# A common assembly module in injectisome and flagellar type III secretion sorting platforms

**DOI:** 10.1038/ncomms8125

**Published:** 2015-05-21

**Authors:** Ryan Q. Notti, Shibani Bhattacharya, Mirjana Lilic, C. Erec Stebbins

**Affiliations:** 1 Laboratory of Structural Microbiology, Rockefeller University, 1230 York Avenue, New York, New York 10065, USA; 2 Tri-Institutional Medical Scientist Training Program, Weill Cornell Medical College, 1300 York Avenue, New York, New York 10021, USA; 3 New York Structural Biology Center, 89 Convent Avenue, New York, New York 10027, USA

## Abstract

Translocating proteins across the double membrane of Gram-negative bacteria, type III secretion systems (T3SS) occur in two evolutionarily related forms: injectisomes, delivering virulence factors into host cells, and the flagellar system, secreting the polymeric filament used for motility. While both systems share related elements of a cytoplasmic sorting platform that facilitates the hierarchical secretion of protein substrates, its assembly and regulation remain unclear. Here we describe a module mediating the assembly of the sorting platform in both secretion systems, and elucidate the structural basis for segregation of homologous components among these divergent T3SS subtypes sharing a common cytoplasmic milieu. These results provide a foundation for the subtype-specific assembly of T3SS sorting platforms and will support further mechanistic analysis and anti-virulence drug design.

## Abstract (web summary)

In bacteria, type III secretion systems (T3SS) allow the direct transport of protein across membranes, and related elements of a sorting platform facilitate hierarchical secretion of protein substrates. Here, the authors illustrate the mechanism of selective assembly of the T3SS sorting platforms in injectisome and flagellar systems.

## Graphical abstract

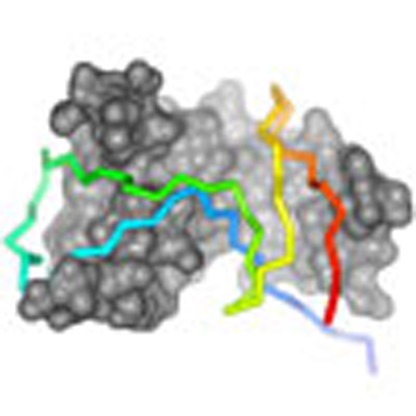

Type III secretion systems (T3SS) allow the transport of protein substrates directly across the double membrane of Gram-negative bacteria. There are two evolutionarily related, yet functionally distinct subtypes of T3SS: ‘injectisomes’, which deliver effector proteins into the cytoplasm of eukaryotic host cells^1^, and the flagellar apparatus, which secretes the polymeric filament used for motility^2^. Despite their functional divergence, injectisomes and the flagella share a common core of homologous gene products and possess ultrastructural similarities^3^. For example, both systems share related elements of a ‘sorting platform’ that facilitates the hierarchical secretion of protein substrates^4^.

Proteomic analyses have identified the major components of the sorting platform for the *Salmonella typhimurium* SPI-1 injectisome: the AAA+ ATPase InvC, its regulator OrgB and the proteins SpaO and OrgA^4^. While SpaO has been shown to be necessary for formation of the sorting platform^4^, little is known about its molecular structure. In *Yersinia*, the SpaO homologue is expressed as a full-length protein as well as a carboxy-terminal fragment translated from an internal translation start site^5^; this carboxy-terminal fragment dimerizes and can interact with the full-length protein. The crystal structure of the *Yersinia* carboxy-terminal dimer is similar to that of its *Pseudomonas*^6^ and flagellar^7^ homologues, together characterizing a structural class known as the surface presentation of antigens (SPOA) domain. Whether other domains within SpaO possess a similar structure, and how these structures correlate with function remains unknown.

In the flagellar apparatus, the SpaO homologues FliM and FliN form a robust, stable ring (the ‘C-ring’) at the cytoplasmic face of the basal body^8^. Electron microscopic analyses have similarly localized the SpaO homologue to the cytoplasmic face of the *Shigella* injectisome^9^, and recent cryoelectron tomographic studies in the same organism identified SpaO homologue-dependent ‘pods’ of density beneath the injectisome^10^. In contrast to the flagellar C-ring, this sub-injectisome structure is less robust^10^, and fluorescence microscopic analysis of the *Yersinia* SpaO homologue show that there is dynamic exchange between cytoplasmic- and injectisome-associated forms^11^. How SpaO and its homologues interact with other elements of the T3SS has yet to be shown at high resolution, and how homologous flagellar and injectisome components are properly segregated to their cognate secretion systems remains an open question.

Here we show that a novel, heterotypic interaction between SPOA domains serves as a scaffold for sorting platform assembly in both injectisome and flagellar T3SS. Solution nuclear magnetic resonance (NMR) data support the crystallographic model, and structure-guided mutagenesis shows that this interaction is necessary for formation of the SpaO–OrgB–InvC complex, the proper localization of SpaO to the bacterial inner membrane and T3SS function. Structures of the flagellar SpaO–OrgB homologues FliM, FliN and FliH reveal a mechanism for the proper segregation of homologous sorting platform components among T3SS subtypes sharing a common cytoplasmic milieu. Together, these structures define a common module utilized in sorting platform assembly and provide insight into the subtype-specific assembly of T3SS.

## Results

### SpaO contains two *bona fide* SPOA domains

**Figure 1:**
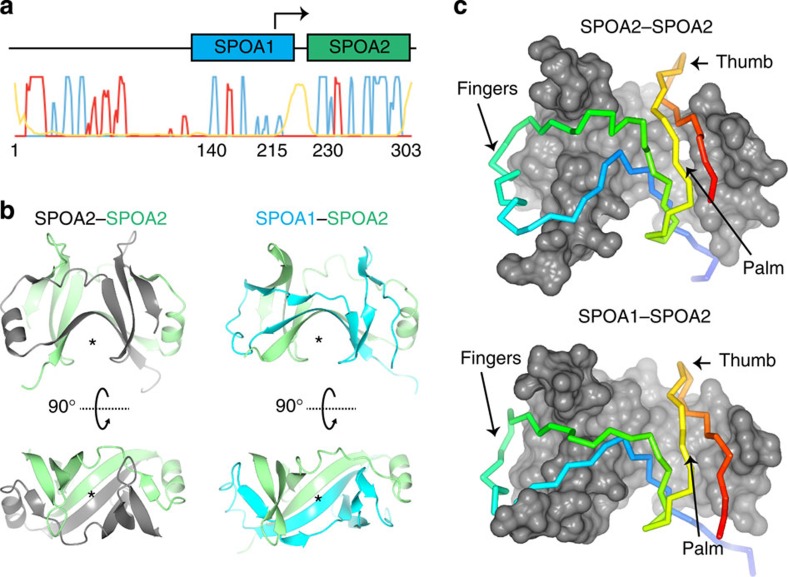
Homotypic and heterotypic SPOA interactions. (**a**) Bioinformatic analysis of SpaO. PSIPRED secondary-structure predictions and sequence homology suggest the presence of two putative SPOA domains in SpaO. Probability of helical character is plotted in red, strand in blue and disorder in yellow. The arrow at codon 203 represents a predicted Val_GTG_ internal translation start site, as has been shown for YscQ Met218 in *Yersinia pseudotuberculosis*^5^. (**b**,**c**) Comparison of homotypic SPOA2–SPOA2 and heterotypic SPOA1–SPOA2 structures from SpaO. (**b**) Ribbon diagrams show the similar organization of secondary-structural elements in both SPOAs. Asterisks denote the antiparallel beta-sheet ‘floor’, (**c**) Amino (blue) to carboxy terminus (red) Cα traces of SPOA2 (top) and SPOA1 (bottom) reveal a similar topology in interaction with SPOA2 (grey surface representation, top and bottom).

To dissect the structural basis for the sorting platform assembly, we determined the structures of individual domains of *S. typhimurium* SpaO and then characterized their interactions with other sorting platform components. Preliminary bioinformatic analyses suggested the presence of two putative SPOA domains in the carboxy-terminal half of SpaO, which we denote SPOA1 and SPOA2 (Fig. 1a). We first determined the structure of the SPOA2 homodimer to 1.35 Å resolution (Fig. 1b,c; Table 1; Supplementary Fig. 1). The SPOA2 homodimer structure is architecturally similar to its homologues^5^,^6^: like two left hands grasping one another, an antiparallel beta-sheet ‘palm’ of each protomer is grasped by the ‘fingers’ of the other, with a ‘thumb’ protruding from the top of the palm and strands from each protomer forming an antiparallel beta sheet on the ‘floor’ of the assembly (Fig. 1b,c). The *Salmonella* SPOA2 homodimer superposes on its *Yersinia* and *Pseudomonas* homologues with 2.24 and 3.05 Å root mean squared deviation (r.m.s.d.), respectively (Supplementary Fig. 1b).

While SPOA1 alone was insoluble, constructs containing both SPOA1 and SPOA2 (residues 140–297) were stable and soluble. SpaO(140–297) was analysed by solution NMR (Supplementary Fig. 2a), and chemical shift deviation (CSD) analysis of backbone amide resonances suggested a secondary-structure pattern similar to that predicted by bioinformatic analyses: two SPOA domains connected by a flexible linker (Supplementary Fig. 2b). We hypothesized that SPOA2 interacts with and stabilizes SPOA1; consistent with this hypothesis, a SPOA1 construct (145–213) could be co-refolded with SPOA2. This complex crystalized, and its structure was determined to 2.9 Å resolution (Fig. 1b,c; Table 1; Supplementary Fig. 3). SPOA1 and SPOA2 form a distinct, heterotypic SPOA–SPOA interaction with an overall topology similar to that of the SPOA2 homodimer. The SPOA1 backbone follows that of the prototypical SPOA fold, retaining the antiparallel beta-sheet floor and fingers-to-palm architecture (Fig. 1b,c). In both SPOA1,2 and the SPOA2 homodimer, interacting protomers each bury about 1,800 Å^2^ against their binding partner. SPOA1,2 and the SPOA2 homodimer superpose with 2.47 Å r.m.s.d. (Supplementary Fig. 3b), and the conformation of SPOA2 in association with SPOA1 is grossly similar to that seen in the homodimer, superposing with an r.m.s.d. of 1.67 Å (Supplementary Fig. 3c).

**Figure 2:**
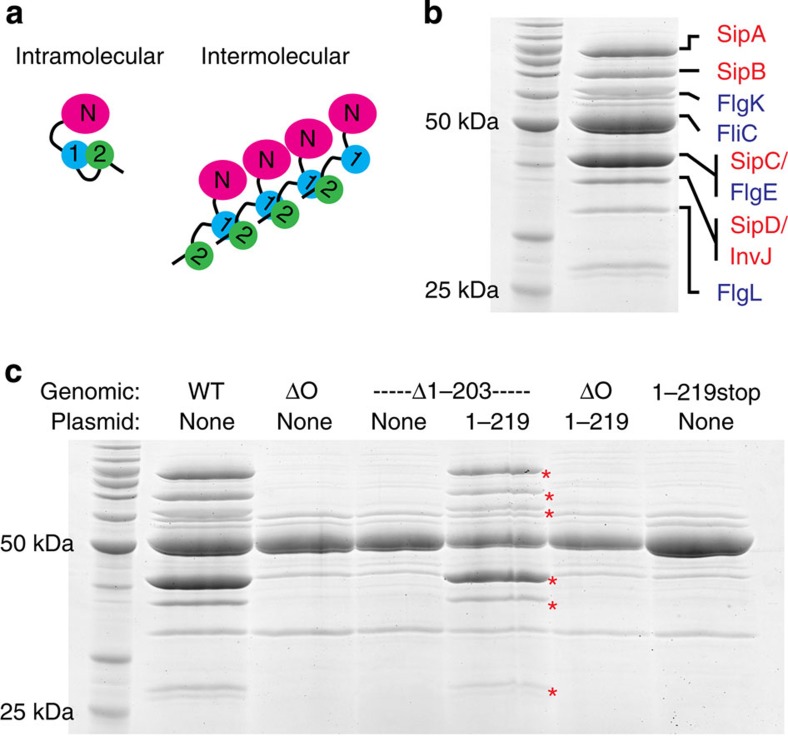
Intermolecular SPOA1–SPOA2 interactions are not necessary for T3SS function. (**a**) Schematic models for putative intra- and intermolecular SPOA1–SPOA2 interactions and their implications for the SpaO oligomerization state. The numbers 1 and 2 indicate the SpaO SPOA1 and SPOA2, respectively, and N indicates the SpaO amino-terminal domain(s). (**b**) Coomassie-stained polyacrylamide gel electrophoresis (PAGE) of *S. typhimurium* culture supernatants grown under T3SS-stimulating conditions (0.3 M NaCl, strain SB1741). Bands previously identified by Mizusaki *et al*.^13^ are noted and colour coded by the T3SS subtype—injectisome in red and flagellar in blue. (**c**) Coomassie-stained PAGE of *S. typhimurium* culture supernatants grown under T3SS-stimulating conditions. Red asterisks indicate injectisome-specific secretion substrates. WT, wild type; ΔO, deletion of *spaO*; Δ1–203, deletion of *spaO* codons 1–203; 1–219, complementation with SpaO(1–219); 1–219stop, insertion of two stop codons following *spaO* codon 219. SpaO was 3 × FLAG tagged at its amino terminus in each *S. typhimurium* strain (except ΔO) and complementation construct. Data shown are representative of three experiments.

Further supporting the hypothesis that SPOA1 and SPOA2 interact in solution, a *post hoc* analysis of the three dimensional ^15^N-edited nuclear Överhauser enhanced spectroscopy-(3D NOESY-HSQC) for SpaO(140–297) revealed long-range amide proton correlations between SPOA1 and SPOA2 (Supplementary Fig. 4). Given the <20 residue linker connecting SPOA1 and SPOA2, they would experience a low millimolar-range relative concentration and would likely interact in an intramolecular fashion (Fig. 2a). However, at high local SpaO concentrations (for example, in association with the T3SS), intermolecular heterotypic SPOA interactions might explain the apparent oligomeric nature of the sorting platform (Fig. 2a). Indeed, a similar model of intermolecular domain swapping was recently suggested for the ring-forming injectisome protein PrgK^12^.

Hypothetical SpaO oligomerization driven by intermolecular heterotypic SPOA interactions would be dependent on the covalent linkage of SPOA1 and SPOA2. Thus, we tested whether genomic deletion of the SpaO amino-terminal domain and SPOA1 can be complemented in trans, as assayed by *Salmonella* T3SS function *in vitro*. When grown under T3SS-stimulating conditions, the culture supernatant of *S. typhimurium* has a stereotyped protein composition, consisting of both flagellar and injectisome secretion substrates (Fig. 2b, secreted proteins are annotated as per Mizusaki *et al*.^13^). Deletion of *spaO* results specifically in the loss of injectisome-dependent secretory products from the culture supernatant, and deletion of *spaO* codons 1–203 phenocopies *spaO* deletion, indicating that the SpaO amino-terminal domain(s) and/or SPOA1 are necessary for T3SS function (Fig. 2c). Because SpaO(1–219) is able to complement the deletion of *spaO* codons 1–203 (Fig. 2c, red asterisks), the covalent linkage of SPOA1 and SPOA2 is not necessary for T3SS function. Thus, if intermolecular heterotypic SPOA interactions do occur *in vivo*, they are not explicitly necessary for secretion. It should be noted that SpaO(1–219) does not complement a full genomic deletion of *spaO*, demonstrating that SPOA2 is also necessary for T3SS function (Fig. 2c). Similarly, insertion of a double stop codon after *spaO* codon 219 abrogates T3SS (Fig. 2c).

### SpaO SPOA1,2 is a scaffold for interaction with OrgB–InvC

**Figure 3:**
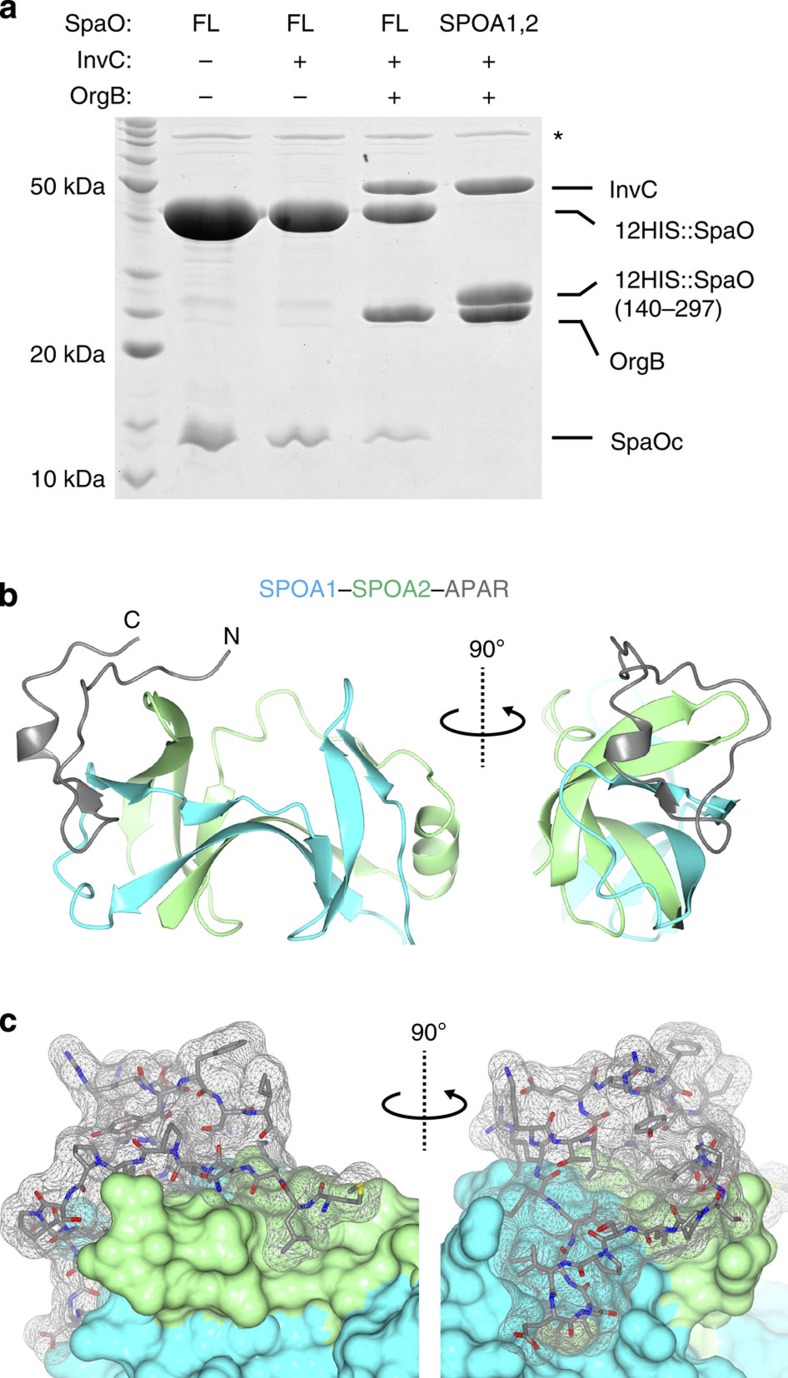
Architecture of the SPOA1,2–APAR interaction. (**a**) Coomassie-stained gel of protein elution from NiNTA resin shows that double-hexahistidine (12HIS)-tagged SpaO (‘FL,’ full length) and SpaO(140–297, Val203_GTT_) are each sufficient to co-affinity purify InvC–OrgB when co-expressed in *Escherichia coli*. Note that OrgB is necessary for ternary complex formation. Asterisk denotes nonspecific co-purifying *E. coli* proteins, likely chaperones. SpaOc indicates the cryptically expressed SPOA2-containing carboxy-terminal fragment. Data shown are representative of three experiments. (**b**) Ribbon diagram of the SpaO–OrgB crystal structure. For simplicity, the T4 lysozyme crystallization chaperone has been omitted and only one of the two constituent complexes from the crystallographic asymmetric unit is shown. The amino and carboxy termini of the OrgB APAR are denoted as ‘N’ and ‘C,’ respectively. (**c**) Surface representation of the complex in **b**. The OrgB APAR (grey mesh) contacts both SpaO SPOA1 (cyan) and SPOA2 (green).

Double-hexahistidine-tagged SpaO is able to co-affinity purify the sorting platform components OrgB and InvC when co-expressed in *Escherichia coli* (Fig. 3a). Formation of the SpaO–OrgB–InvC termary complex is OrgB dependent, as SpaO alone is insufficient to co-affinity purify InvC (Fig. 3a). We hypothesized that SPOA1,2 might serve as a scaffold for the interaction of SpaO with OrgB–InvC. Indeed, SPOA1,2 is sufficient to co-affinity purify OrgB–InvC (Fig. 3a). This construct contains a Val203_GTG_-to-Val203_GTT_ mutation to prevent the duplicitous translation of SPOA2 from its cryptic internal translation start site, demonstrating that the SPOA2 homodimer is dispensable for SpaO–OrgB–InvC complex formation.

OrgB and its homologues are predicted to share a common amino-terminal organization: a disordered region followed by a coiled coil. In the flagellar system, the unstructured region at the amino terminus of the OrgB homologue FliH is necessary for its interaction with the SpaO homologues FliM and FliN^14^. We solublized the pre-coiled-coil region of OrgB (residues 1–30) by genetic fusion to T4 lysozyme and found that it bound to SpaO SPOA1,2. Herein, we will refer to the SPOA1,2-binding region at the amino terminus of OrgB and its homologues as the adaptor peptide of the ATPase regulator (APAR).

The SPOA1,2–OrgB(APAR)::lysozyme complex was crystalized and its structure solved to 2.0 Å resolution (Fig. 3b; Table 1; Supplementary Figs 5 and 6). The OrgB APAR forms a lariat-like structure, contacting the thumb of SPOA2 and fingers of SPOA1 (Fig. 3b,c). OrgB makes substantial contact with both SPOA1 and SPOA2 of SpaO, burying 570 Å^2^ against SPOA1 and 470 Å^2^ against SPOA2. In the APAR-bound structure, there is little change in the conformation of SpaO (Supplementary Fig. 5d, 1.01 Å r.m.s.d.).

**Figure 4:**
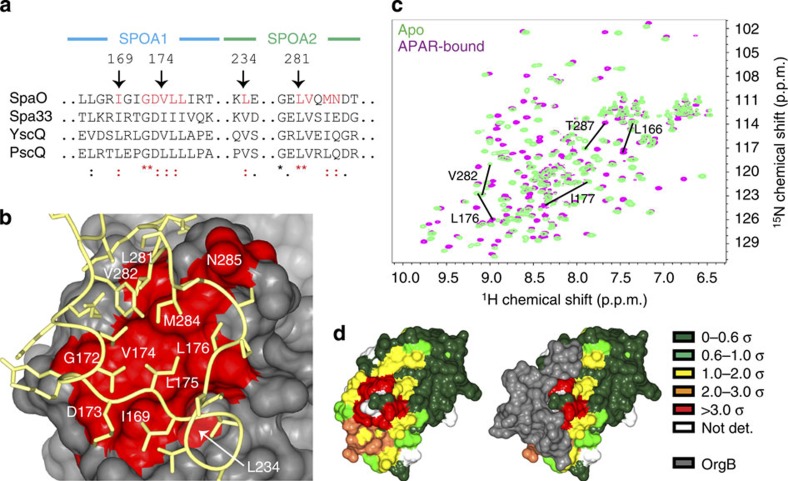
The APAR-binding site of SpaO. (**a**) The SpaO residues at the APAR interaction site are highly conserved across homologues in other species. Excerpts of the M-COFFEE alignment of SpaO, *Shigella flexneri* Spa33, *Yersinia enterocolica* YscQ and *Pseudomonas aeruginosa* PscQ are shown with conserved APAR-interacting residues highlighted in red. Symbols beneath the alignment indicate the degree of conservation: asterisks denote full conservation, colons denote strong similarity, and dots denote weak similarity. (**b**) A surface representation of SpaO with the conserved interfacial residues identified in **a** are coloured red and the OrgB APAR backbone is yellow. (**c**) Overlayed ^15^N-heteronuclear single quantum coherence spectra of apo- (green) and APAR-bound (violet) SpaO(140–297). The five largest peak shifts are noted. (**d**) The solution interaction data from **c** are mapped onto the SpaO–OrgB crystal structure. Surface residues are colour coded by the size of their weighted CSD in units of s.d. Residues not assigned an amide resonance in one of the two data sets are left white. The same view of SpaO is shown with and without OrgB (grey surface).

**Figure 5:**
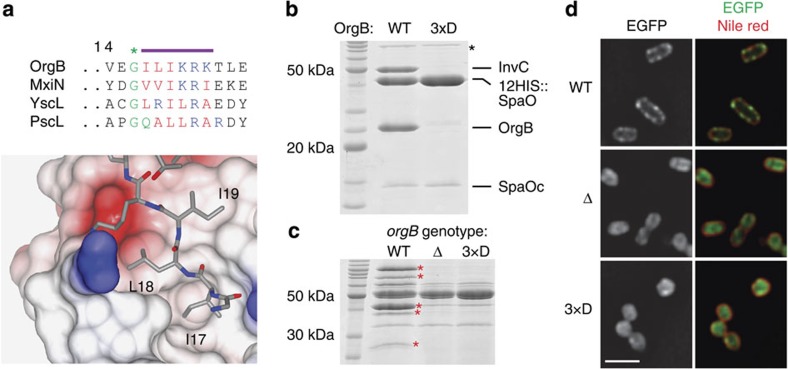
Structure-guided disruption of the SPOA1,2–APAR interaction. (**a**) Clustal Omega alignment of the APAR regions of OrgB, *S. flexneri* MxiN, *Y. enterocolica* YscL and *P. aeruginosa* PscL. The conserved pseudo-lariat apex glycine is indicated by a green asterisk and the subsequent patch of aliphatic (red) and basic (blue) amino acids is highlighted with a purple bar. Beneath, the binding site for OrgB(17–19) (grey) is shown as an electrostatic surface. OrgB(1–15) have been removed for clarity. (**b**) Coomassie-stained polyacrylamide gel electrophoresis of the protein elution from NiNTA resin shows that double-hexahistidine-tagged SpaO can co-affinity purify wild-type InvC–OrgB but not InvC–OrgB(I17D,L18D,I19D) when co-expressed in *E. coli.* 3 × D indicates the OrgB(I17D,L18D,I19D) triple mutant. Asterisk denotes nonspecific co-purifying *E. coli* proteins, likely chaperones. SpaOc indicates the cryptically expressed SPOA2-containing carboxy-terminal fragment. (**c**) Coomassie-stained culture supernatant from wild-type (WT, strain SB1741), *orgB* deletion(Δ) and *orgB(I17D*,*L18D*,*I19D)* (3 × D) *S. typhimurium* shows loss of injectisome substrate (red asterisks) secretion in the mutants, while flagellar secretion remains intact. (**d**) Widefield microscopic imaging of fixed *S. typhimurium* shows exclusive perimembranous localization of SpaO in the WT background, but cytoplasmic localization in the *orgB* mutants (scale bar, 2 μm, single z-slices shown). Data shown in **b**–**d** are representative of three experiments.

Independent NMR analyses of SpaO(140–297) bound to OrgB(APAR) in solution are consistent with the interface defined in the crystal (Fig. 4). Compared with apo-SpaO, the largest CSDs of backbone amide resonances in SpaO–OrgB map on the crystal structure to residues involved in the interface, which are highly conserved across both the *Salmonella*/*Shigella* and *Yersinia*/*Pseudomonas* clades (Fig. 4; Supplementary Fig. 7). In the crystal, these residues form the docking site for OrgB residues Ile17, Leu18 and Ile19 (Fig. 5a). The OrgB surface area buried by these three residues (360 Å^2^) accounts for approximately one-third of the APAR’s total buried area. Here, the APAR shows noteworthy sequence homology: immediately following a conserved glycine (Gly16, pseudo-lariat apex) is a string of aliphatic and basic amino acids in each homologue (Fig. 5a).

### The SPOA1,2–APAR interaction is necessary for T3SS function

To test whether the SPOA1,2–APAR interaction *per se* is necessary for T3SS function, we constructed an OrgB triple mutant (I17D,L18D,I19D) to disrupt its interaction with SpaO. As predicted, SpaO failed to co-affinity purify OrgB(I17D,L18D,I19D)–InvC when co-expressed in *E. coli* (Fig. 5b), and the aspartate triple mutation completely abolished T3SS *in vivo* (Fig. 5c). Fluorescence microscopic analyses of the *Yersinia* SpaO homologue have shown it to localize in discrete perimembranous punctae^11^. Might the SPOA1,2–APAR interaction function to localize SpaO to the bacterial inner membrane? In an otherwise wild-type genomic background, an EGFP::3 × FLAG::SpaO fusion exhibits punctate, perimembranous localization, consistent with its recruitment to injectisome basal body channels (Fig. 5d). Deletion of *orgB* disrupts proper SpaO localization, producing a more diffuse, cytoplasmic pattern, and the asparate triple mutation was sufficient to phenocopy the *orgB* deletion mutant (Fig. 5d). Together, these data suggest that the SpaO(SPOA1,2)–APAR assembly is necessary for the proper localization of SpaO to discrete perimembranous puncta, and that this arrangement is required for T3SS function.

### A divergent SPOA1,2–APAR assembly in the flagellar T3SS

**Figure 6:**
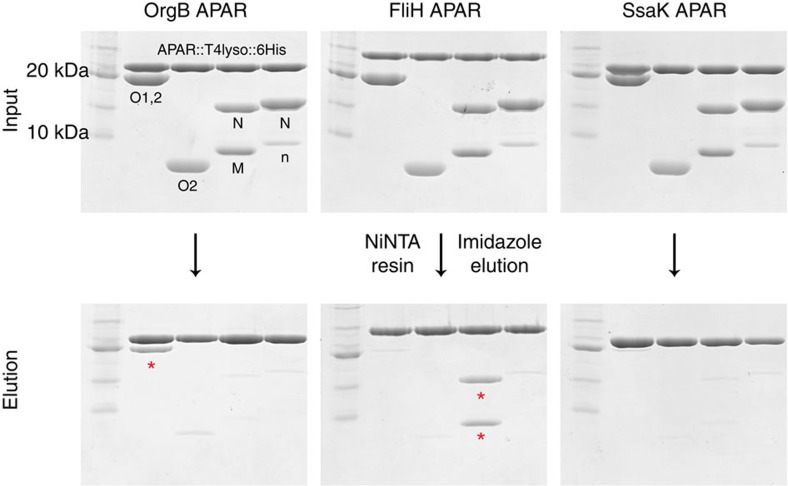
APARs preferentially interact with their cognate SPOA1,2. Coomassie-stained gels showing the input and imidazole elution for APAR–SPOA co-affinity purification experiments. Red asterisks indicate the cognate SPOA1,2 band(s). T4lyso, T4 lysozyme; O1,2, SpaO(140–297); O2, SpaO(232–297); M, FliM(245–320); N, FliN (1–137); n, co-purifying amino-terminal FliN degradation product. Data shown are representative of three experiments.

The flagellar C-ring is primarily composed of three proteins: FliM, FliN and FliG^8^. The SpaO homologues FliM and FliN are predicted to contain one SPOA domain each, which we designate as SPOA1 and SPOA2, respectively. Paralleling the injectisome, FliN is known to interact with the OrgB homologue FliH^14^. The evolutionary relationship between injectisomes and flagella creates a practical conundrum: how are homologous T3SS components segregated to their corresponding secretion systems within a common cytoplasmic milieu? To qualitatively assess the subtype specificity of SPOA–APAR interactions, we co-affinity purified a panel of *Salmonella* SPOA domains with hexahistidine-tagged APAR::lysozyme fusions (Fig. 6). Indeed, the OrgB and FliH APARs robustly co-affinity purify their cognate SPOA1,2 but not that of the other T3SS subtype (Fig. 6, red asterisks). Neither SpaO nor FliM–FliN are pulled down by the APAR from a second pathogenic T3SS found in *S. typhimurium* (SPI-2 SsaK). Consistent with the observation that the OrgB APAR interacts with surfaces on both SPOA1 and SPOA2, the OrgB and FliH APARs more robustly pull down their cognate SPOA1,2 than homodimeric SPOA2 (Fig. 6).

We hypothesized that divergence of the SPOA1,2–APAR assembly architecture contributes to proper component segregation among T3SS subtypes, and sought to structurally characterize the flagellar SPOA–APAR interactions. While complexes of FliM and FliN were stable, they were resistant to crystallization. Interestingly, FliM and FliN can be fused and still support flagellin secretion (Supplementary Fig. 8a) and some swarming motility^15^. We crystallized the SPOA of FliM (residues 245–334) fused to FliN(5–137), and its structure was solved to 2.56 Å (Table 2; Supplementary Fig. 8). Architecturally, the FliM(SPOA1)–FliN(SPOA2) interaction is similar to that of SpaO (Supplementary Fig. 8e, 2.28 Å r.m.s.d.), with the exception of additional helices present at the carboxy terminus of each SPOA, as observed in FliN homodimers from *Thermotoga maritima*^7^. The similarity of these structures is consistent with the SPOA heterotypic interaction being generalizable across T3SS subtypes.

**Figure 7:**
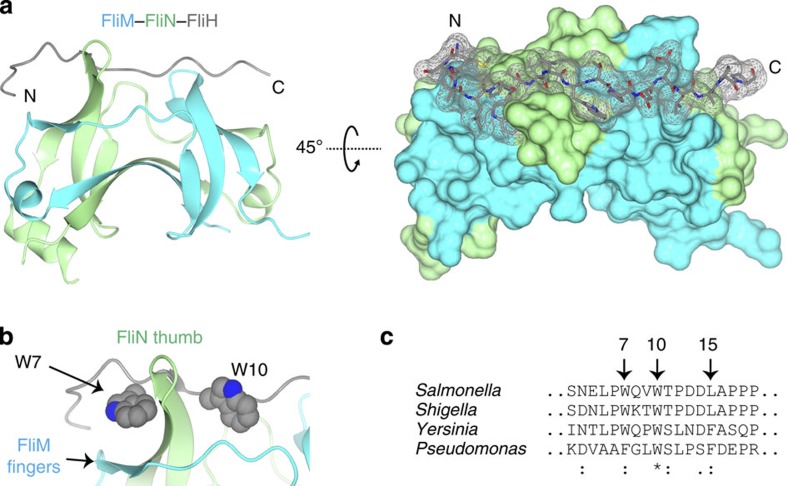
Structure of the SPOA1,2–APAR interaction in the flagella. (**a**) Ribbon diagram (left) and surface representation (right) of the FliM–FliN–FliH structure. T4 lysozyme has been omitted. N and C indicate the amino and carboxy termini of the FliH APAR, respectively. (**b**) A zoomed view of the FliH aromatic clamp, with the side-chain atoms of FliH W7 and W10 represented as spheres. (**c**) Excerpted M-COFFEE alignment of FliH with its homologues from *S. flexneri*, *Y. enterocolica* and *P. aeruginosa*. Highly conserved residues of interest are noted (*S. typhimurium* numbering). Symbols beneath the alignment indicate the degree of conservation: asterisks denote full conservation, colons denote strong similarity, and dots denote weak similarity.

To elucidate the mechanism of FliH-specific assembly with FliM–FliN, we co-crystalized the FliM(SPOA)::FliN fusion with a FliH(1–18)::lysozyme fusion (Fig. 7; Table 2; Supplementary Fig. 9). As with its injectisome counterparts, the FliM–FliN SPOA1,2 did not undergo large conformational changes upon APAR binding (Supplementary Fig. 9c, 1.11 Å r.m.s.d.); however, the binding mode for the FliH APAR is radically different. In contrast to the OrgB pseudo-lariat, the FliH APAR adopts a near-linear conformation along the ‘top’ of FliM–FliN (Fig. 7a). As observed in the SpaO–OrgB assembly, the FliH APAR makes extensive contact with both SPOA1 and SPOA2 (Fig. 7a), supporting the observation that the FliH APAR interacts more strongly with the FliM–FliN heterodimer than the FliN homodimer (Fig. 6).

The FliH–FliM–FliN assembly is characterized by the burial of several highly conserved hydrophobic FliH side chains. Two tryptophan side chains form an aromatic clamp, which binds hydrophobic pockets on opposite faces of the FliN thumb (Fig. 7b). These residues are critical for flagellar function^14^ and are highly conserved (Fig. 7c). Similarly, the bulky side chain of FliH Leu15 fills a hydrophobic pocket on the thumb of FliM (Supplementary Fig. 10). The binding interfaces for these three residues are formed by both FliM and FliN and are highly conserved across species (Supplementary Fig. 10). This structure presents a conserved model for FliH–FliM–FliN interaction, which is distinct from that of SpaO–OrgB.

## Discussion

We present here a series of structures that yield critical mechanistic insights into T3SS sorting platform assembly across multiple species and secretion subtypes. The existence of heterotypic SPOA interactions provides a structural explanation for the observed ∼1:3 stoichiometry of SPOA1 to SPOA2 in SpaO homologues^5^. While two of these SPOA2 domains could be accounted for by a homodimer interacting with full-length SpaO, the conformation of the third SPOA2 (located in the full-length protein) was unclear. Previous reports had proposed the existence of an alternate autostabilizing conformation for the third SPOA2 (ref. 5). We show here that the third SPOA2 can be stabilized by a SPOA1–SPOA2 interaction.

Similar to SpaO and its injectisome homologues, the ratio of FliM to FliN *in situ* is estimated to be 1:3 (ref. 8). In the context of our FliM–FliN structure, this suggests a model for FliM–FliN interaction similar to that of SpaO. FliM(SPOA1) would engage FliN(SPOA2) in a heterotypic SPOA–SPOA interaction, and additional homodimeric FliN would interact with FliM–FliN in an as of yet undetermined fashion (analogous to the SpaO SPOA2 homodimer interaction with full-length SpaO). However, reports of FliN tetramerization and FliM:FliN ratios between 1:3 and 1:4 suggests that more complicated higher-order structures may be used by the flagellar apparatus^7^. It should also be noted that while previous investigations of the flagellar T3SS have focused on the interaction between FliH and FliN specifically^14^, our structures and biochemical data show that the FliH APAR more strongly interacts with the FliM–FliN complex than with FliN alone, suggesting that the FliM–FliN complex is the physiologically relevant binding partner for FliH.

**Figure 8:**
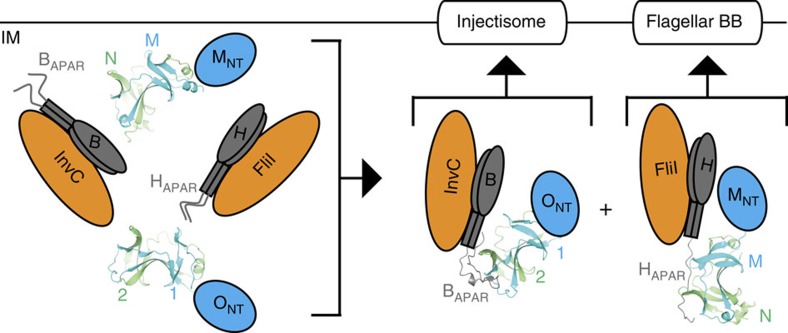
Subtype-specific assembly of the T3SS sorting platform by SPOA1,2–APAR interactions. Schematic illustration of the proposed role for the SPOA1,2–APAR assembly in organizing and localizing the T3SS sorting platforms in a subtype-specific fashion. IM indicates the inner membrane; O_NT_, the SpaO amino-terminal domain(s); 1 and 2, SpaO SPOA1 and SPOA2; B, OrgB; H, FliH; M_NT_, the FliM amino-terminal domains; M and N, the SPOA domains of FliM and FliN; injectisome, the membrane integral components of the pathogenic T3SS; flagellar BB, the flagellar basal body and associated integral membrane components.

Our structures suggest a partial model for the subtype-specific assembly of the T3SS sorting platforms: the heterotypic interaction between SPOA domains within a given T3SS subtype functions as an adaptor for ATPase and its regulator through interaction with the APAR peptide (Fig. 8). However, a number of questions remain regarding the higher-order architecture of the sorting platform *in situ.* We hypothesize that the puncta formed by SpaO *in vivo* represent the high-molecular weight sorting platforms described by Lara-Tejero *et al*.^4^. Diepold *et al*. have quantified the stoichiometry and dynamics of these puncta in *Yersinia*, showing them to possess ∼22 copies of the SpaO homologue per punctum and to be in dynamic exchange with the cytoplasm^11^. In contrast, the recent tomographic reconstruction of *Shigella* injectisomes by Hu *et al*. revealed the presence of only six SpaO homologue-dependent pods of density beneath the injectisome, and their localization was OrgB homologue independent^10^. Taken together with our findings, these results suggest that there may be two subpopulations of SpaO *in vivo*: one stably associated with the injectisome, and a second dynamic population in exchange with the cytoplasm, requiring the SPOA1,2–APAR interaction to form high-molecular weight, perimembranous sorting platforms. Recent analyses of FliI ATPase dynamics by Bai *et al*. suggest a similar two-population model, which they hypothesize functions to deliver secretion substrates to the assembling flagella^16^.

What might be the mechanism for sorting platform targeting, and how might this factor into T3SS machine function? Perhaps APAR binding to the SPOA1,2 scaffold induces conformational changes in OrgB, InvC and/or the amino terminus of SpaO, which facilitates interaction with the membrane integral components of the T3SS. Alternatively, the SPOA1,2–APAR assembly might function simply by inducing proximity between sorting platform components. Intriguingly, the FliH APAR region has previously been shown to interact with the membrane integral export gate protein FlhA^17^, suggesting that OrgB/FliH may function as a hub, bridging the ATPase, export gate and SpaO/FliM–FliN. How the sorting platform component OrgA—which lacks a clear flagellar homologue—factors into complex assembly remains to be determined. Similarly, the implications of sorting platform assembly for substrate recruitment, dechaperoning and secretion remain unclear. Our structures will support the precise interrogation of sorting platform interactions in the biomechanics of secretion, and the necessity of the SPOA1,2–APAR interaction makes it a novel target for the design of anti-virulence compounds.

## Methods

### Bioinformatics

Sequence alignments were performed using Clustal Omega^18^ or M-COFFEE^19^. Secondary-structure and disorder predictions were performed using the PSIPRED server^20^.

### Molecular biology

PCR was performed using OneTaq (New England Biolabs), Phusion (New England Biolabs) or PfuTurbo (Agilent) as per manufacturer guidelines with oligonucleotides purchased from Integrated DNA Technologies. All mutations or gene fusions were created by overlap extension PCR. Gene sequences from *S. typhimurium* were PCR amplified from the T3SS-competent strains SB300 (wild type; gift from J. Galán) or SB1741 (3 × FLAG::SpaO, silent SpaO L79_CTG_ to L79_CTA_ variant; gift from J. Galán)^4^. The T4 lysozyme (C54T, C97A) sequence was obtained from Addgene plasmid 18111. An additional mutation (D20N) in T4 lysozyme was made to decrease toxicity in *E. coli*^21^, and the terminal three residues were mutated to alanines to decrease conformational entropy. Standard molecular biology protocols were followed to clone sequences of interest into modified pCDFduet or pETduet vectors for expression in *E. coli* or pBAD for expression in *S. typhimurium*. Restriction enzymes and Quick Ligase (New England Biolabs) were used as per manufacturer specifications. *Salmonella* genomic mutants were produced using homologous recombination from SacB-expressing suicide plasmids^22^. All SpaO and OrgB mutants were prepared on the SB1741 background. FliM and FliN mutants were prepared on the SB300 background.

### Protein expression and purification

Constructs were transformed into BL21(DE3)Gold *E. coli* for heterologous expression and protein expression induced mostly as described^23^. Specifically, bacteria were grown to an optical density at 600 nm of 0.5–0.6 at 37 °C in LB medium, the cultures were cooled to 18 °C, induced with 250 μM isopropyl-b-D-thiogalactoside and grown overnight at 18 °C. Selenomethionine (SeMet)-substituted protein was produced in the methionine auxotrophic *E. coli* B834(DE3) grown in methionine-free media supplemented with SeMet^23^. Uniformly labelled ^15^N/^13^C or ^2^H/^15^N/^13^C protein samples were produced by overexpression in isotopically enriched minimal media. Deuterium oxide, ^15^N-ammonium chloride, and ^13^C-glucose were obtained from Cambridge Isotope Labs.

After induction overnight at 18 °C, cells were harvested by centrifugation and resuspended in lysis buffer (200 mM NaCl, 20 mM Tris-Cl pH=8.0, 5% v/v glycerol, 3 mM imidazole-Cl pH=8.0, 5 mM MgCl_2_, 5 mM 2-mercaptoethanol, 1 mM phenylmethylsulphonyl fluoride and 0.1 mg ml^−1^ DNaseI). Cells were lysed by 1–2 passes through a mechanical homogenizer (Avestin C5) at 4 °C.

Proteins were purified from *E. coli* cell lysates under native or denaturing conditions (as indicated for each downstream application below) and affinity purified on NiNTA resin (Qiagen). For purification under native conditions, all steps were performed at 4 °C. Lysate was clarified by centrifugation for 30 min at 30,000*g* and loaded onto NiNTA resin by gravity flow. The column was washed with 5–10 volumes of wash buffer (200 mM NaCl, 20 mM Tris-Cl pH=8.0, 5% v/v glycerol, 30 mM imidazole-Cl pH=8.0) and then eluted in elution buffer (200 mM NaCl, 20 mM Tris-Cl pH=8.0, 5% v/v glycerol, 360 mM imidazole-Cl pH=8.0). The elution was supplemented with 1 mM EDTA and dialyzed overnight against 200 mM NaCl, 20 mM Tris-Cl pH=8.0 and 1 mM dithiothreitol. Affinity tags were removed by cleavage with HRV 3C protease.

For purification under denaturing conditions, guanidinium chloride was added to the lysate to a final concentration of 6 M. The post-extraction lysate was clarified by centrifugation at 30,000*g* for 15 min at 4 °C and loaded onto NiNTA resin in a batch at 25 °C. Still at 25 °C, the resin was washed with denaturing wash buffer (8 M urea, 500 mM NaCl, 20 mM Tris-Cl pH=8.0 and 30 mM imidazole-Cl pH=8.0) and eluted in denaturing elution buffer (8 M urea, 200 mM NaCl, 20 mM Tris-Cl pH=8.0 and 360 mM imidazole-Cl pH=8.0). The elution was supplemented with 5 mM EDTA and 5 mM dithiothreitol (DTT), and the protein refolded by dialysis against 200 mM NaCl, 20 mM Tris-Cl pH=8.0 and 1 mM DTT (3–4 changes, dialysis time of 24 h total, 4 °C). For T4 lysozyme fusions, Hepes-Na pH=7.0 was substituted for Tris-Cl pH=8.0. Insoluble material was removed by centrifugation or filtration and affinity tags were removed by cleavage with HRV 3C protease.

Affinity-purified proteins were further purified by ion-exchange chromatography using an AKTA FPLC and the following columns (GE Healthcare): T4 lysozyme fusions were purified by cation exchange on a SourceS column; all other constructs were purified by anion exchange on a SourceQ column. For cation-exchange chromatography, proteins were loaded in a batch in 10 mM Hepes-Na pH=7.0, 50–100 mM NaCl and eluted by a NaCl gradient (from 0 to 1,000 mM) in the same buffer. For anion exchange, proteins were loaded in a batch in 20 mM Tris-Cl pH=8.0, 50–100 mM NaCl and eluted by a NaCl gradient (from 0 to 1,000 mM) in the same buffer.

Prior to crystallography, ion-exchange-purified proteins were further purified by gel filtration chromatography on a Superdex 75 column (GE Healthcare) in final buffer (200 mM NaCl, 20 mM Tris-Cl pH=8.0 and 2 mM DTT) and concentrated using centrifugal concentrators (Amicon). To form the SpaO–OrgB::lysozyme complex for crystallization, cation-exchange-purified OrgB(1–30)::T4 lysozyme was mixed with an excess of anion-exchange-purified SpaO(145–213)+SpaO (232–297) and allowed to incubate overnight at 4 °C. The SpaO–OrgB::lysozyme complex was then purified by gel filtration chromatography. To form the FliM::FliN–FliH::lysozyme complex for crystallization, anion-exchange-purified FliM(245–334)::FliN(5–137) was mixed with an excess of cation-exchange-purified FliH(1–18)::T4 lysozyme and allowed to incubate overnight at 4 °C. The FliM::FliN–FliH::lysozyme complex was then purified by gel filtration chromatography.

### Crystallization

All proteins were crystallized by hanging-drop vapour diffusion with 1:1 and 2:1 ratios of protein (in final buffer) to precipitant at 25 °C (except where noted). For crystallization, SpaO(232–297) and FliM(245–334)::FliN(5–137) were purified under native conditions; SpaO(145–213) and SpaO (232–297) were purified under denaturing conditions and co-refolded; the T4 lysozyme fusions were purified under denaturing conditions, refolded and mixed with their cognate SPOA1,2 as described above. The protein concentrations, crystallization buffers and cryoprotection conditions for each protein or complex are as follows:

SpaO(232–297) was concentrated to 8 mg ml^−1^ and crystallized with 35% PEG400, 200 mM calcium acetate and 100 mM sodium acetate pH=5.0. Crystals were cryoprotected in the mother liquor. Microseeding was performed to enhance crystal uniformity and diffraction. Briefly, crystals to be seeded were harvested in precipitant solution and vortexed in a microfuge tube with a small stir bar for ∼60 s. The slurry of microseeds was serially dilluted (5–10-fold steps) in precipitant solution and five selected microseed precipitant mixtures were mixed with fresh protein as in a normal hanging-drop experiment.

SpaO(145–213)+SpaO(232–297) was concentrated to 12 mg ml^−1^ and crystallized with 25% PEG400, 10% isopropanol and 100 mM sodium citrate pH=5.6 at 4 °C. Microseeding (as above) was performed to enhance crystal uniformity and diffraction. Crystals were cryoprotected in mother liquor with the PEG400 concentration raised to 37.5%.

SpaO(145–213)+SpaO(232–297)+OrgB(1–30)::T4 lysozyme was concentrated to 18.5 mg ml^−1^ and crystallized with 25% PEG3350, 200 mM ammonium formate and 100 mM sodium acetate pH=5.0. Microseeding (as above) was performed to enhance crystal uniformity and diffraction. Crystals were cryoprotected in 30% PEG3350, 10% glycerol, 200 mM ammonium acetate and 100 mM sodium acetate pH=5.0.

SpaO(145–213, SeMet)+SpaO(232–297, SeMet)+OrgB(1–30)::T4 lysozyme (native) was concentrated to 18 mg ml^−1^, supplemented with 50 mM maltose and crystallized with 25% PEG3350, 200 mM ammonium formate and 100 mM sodium acetate pH=5.0. Microseeding (as above) was performed to enhance crystal uniformity and diffraction. Crystals were cryoprotected in 25% PEG3350, 10% ethylene glycol, 200 mM ammonium formate, 100 mM sodium acetate pH=5.0 and 50 mM maltose.

FliM(245–334)::FliN(5–137) was concentrated to 7.5 mg ml^−1^ and crystallized with 2.2 M NaCl and 100 mM imidazole-Cl pH=8.0. Crystals were cryoprotected with 2 M NaCl, 100 mM imidazole-Cl pH=8.0 and 30% glycerol.

FliM(245–334)::FliN(5–137)+FliH(1–18)::T4 lysozyme was concentrated to 17 mg ml^−1^ and crystallized with 11% PEG400 and 100 mM sodium potassium phosphate pH=6.5. Crystals were cryoprotected with 40% PEG400 and 200 mM sodium potassium phosphate pH=6.5.

### Structure determination

Data were collected at the National Synchrotron Light Source (Brookhaven National Laboratory) beamline X29A at a temperature of −173 °C using the following X-ray wavelengths: 0.979 Å for SeMet crystals, 1.075 Å for native crystals. Diffraction data sets were indexed and integrated in iMOSFLM^24^ and scaled and reduced with AIMLESS^25^. Data sets were truncated at *I*/*σI*>2.0, and all sets were determined to have a CC_1/2_>0.7 in the outermost resolution shell^26^ (Tables 1 and 2).

The PHENIX program suite^27^ was used to solve the crystallographic phase problem. SpaO(232–297), SpaO(145–213)+SpaO(232–297) and FliM(245–334)::FliN(5–137) were solved by SeMet single-wavelength anomalous diffraction in Autosol. The SPOA1,2–APAR::lysozyme structures were solved by molecular replacement in Phaser-MR using the experimentally phased cognate SPOA1,2 structure and T4 lysozyme (PDB 2LZM). Structures were built in Phenix (Autobuild) with additional manual model building performed in Coot^28^.

Structures were refined and validated in Phenix (Tables 1 and 2). SpaO(145–213)+SpaO(232–297)+OrgB(1–30)::T4 lysozyme crystals exhibited twinning and were refined in Phenix using the twin law l,−k,h. Ramachandran statistics for all models are as follows: SpaO(232–297, SeMet): 98% favoured, 0% outliers; SpaO(145–213)+SpaO(232–297): 89% favoured, 3% outliers; SpaO(145–213)+SpaO(232–297)+OrgB(1–30)::T4 lysozyme: 94% favoured, 0.8% outliers; SpaO(145–213, SeMet)+SpaO (232–297, SeMet) +OrgB(1–30)::T4 lysozyme: 89% favoured, 1.8% outliers; FliM(245–334)::FliN(5–137, SeMet): 92% favoured, 0.9% outliers; FliM(245–334)::FliN(5–137)+FliH(1–18)::T4 lysozyme: 94% favoured, 0.9% outliers.

ANODE^29^ was used to perform *post hoc* analysis of anomalous scatters in SpaO(145–213, SeMet)+SpaO(232–297, SeMet)+OrgB(1–30)::T4 lysozyme crystals, providing additional empirical support for the SpaO–OrgB model coordinates (Supplementary Fig. 6b). Except where indicated, all representations of models and maps for figures were produced in QtMG^30^.

### NMR spectroscopy

The NMR sample of refolded SpaO(140–297) consisted of 0.3 mM U-^2^H/^15^N/^13^C-labelled protein in 10 mM citrate buffer at pH 5.6 with 90% H_2_O/10% D_2_O (v/v), 100 mM NaCl and 1 mM dithiothreitol. For comparison of the apo and APAR-bound forms, ^15^N/^13^C-labelled SpaO(140–297) was co-refolded with an excess of unlabelled thioredoxin::OrgB(2–30). The thioredoxin solublization tag was cleaved off by overnight incubation with HRV 3C protease. Protease and affinity tags were removed on NiNTA resin and the SpaO–OrgB complex was separated from the majority of free thioredoxin by Superdex 75 gel filtration chromatography. The final concentration of the protein complex was 0.2 mM in 10 mM citrate buffer at pH 5.6 supplemented with 10% v/v deuterium oxide, 100 mM NaCl and 2 mM dithiothreitol.

The NMR data were collected on Bruker 600, 800 and 900 MHz AVANCE spectrometers equipped with TCI/TXI CryoProbes at 20 °C for the apo-SpaO and 30 °C for the APAR-bound forms. For resonance assignments of apo-SpaO, transverse relaxation-optimized triple-resonance^31^ experiments including trHNCO, trHN(CA)CO, trHNCA, trHN(CO)CA, trHNCACB and trHN(CO)CACB were acquired at 600 and 900 MHz. A three dimensional ^15^N-edited nuclear Överhauser enhanced spectroscopy-heteronuclear single quantum coherence spectrum with 100 ms mixing time was also acquired at 900 MHz. To assign APAR-bound SpaO, a suite of conventional backbone experiments^32^ were acquired at 600 and 800 MHz.

The data were processed in Topspin 2.1 spectra and analysed using the Autolink module in CARA 1.5 (ref. 33). In both apo-SpaO and its complex with APAR, we were able to successfully assign >95% of the backbone resonances. The heteronuclear chemical shifts were analysed using the TALOS+^34^ database to predict the secondary structure of the protein. The weighted CSDs were calculated from amide proton (H) and nitrogen chemical shifts (^15^N) using the following equation: CSD=√((Δ*δ*H)^2^+((Δ*δ*^15^N)/5)^2^)

### Co-affinity purification assay

For co-affinity purification of the SpaO–OrgB–InvC complex (Figs 3 and 5), the proteins indicated were co-expressed and purified under native conditions as described above. For the SPOA–APAR::lysozyme pull-down experiment (Fig. 6), the indicated SPOA-containing proteins were Ni-affinity purified under native conditions, their affinity tags were removed by overnight incubation with HRV protease 3C and they were further purified by anion-exchange chromatography (as above). APAR::lysozyme fusions were separately purified under denaturing conditions and were subjected to cation-exchange chromatography after refolding (as above). One mg of hexahistidine-tagged APAR::lysozyme fusion protein was mixed with 2 mg of the indicated SPOA-containing protein in 0.2 M NaCl and 20 mM Tris-Cl pH=8.0 (final volume 4 ml) and incubated on ice for 2 h. The mixture was passed twice over 2 ml of NiNTA resin, washed with 8 ml wash buffer (200 mM NaCl, 20 mM Tris-Cl pH=8.0, 5% v/v glycerol and 30 mM imidazole-Cl pH=8.0) and then eluted in 3.5 ml elution buffer (200 mM NaCl, 20 mM Tris-Cl pH=8.0, 5% v/v glycerol and 360 mM imidazole-Cl pH=8.0).

### *In vitro* secretion assay

*S. typhimurium* of the indicated genotype were grown for 6 h at 37 °C in LB medium with NaCl supplemented to a final concentration of 0.3 M. Cells were pelleted by centrifugation at 3,400*g* for 0.5–1 h and the supernatants were 0.22-μm filtered. Secreted proteins were precipitated from the filtered supernatants with 15% trichloroacetic acid overnight at 4 °C. The precipitate was pelleted by centrifugation at 3,400*g* for 1 h at 4 °C, resuspended in ice-cold acetone and transferred to a microfuge tube. After 0.25 h on ice, the precipitate was harvested by centrifugation at 16,000*g* for 0.75 h at 4 °C and resuspended in 0.2 M Tris-Cl pH=8.0 and 0.2 M NaCl to neutralize any residual acid before the addition of SDS–polyacrylamide gel electrophoresis loading buffer. For plasmid complementation analysis, *S. typhimurium* were electroporated with SpaO sequences cloned into the pBAD vector and expression was induced with 0.01% arabinose for the entire duration of the experiment.

### Fluorescence microscopy

*S. typhimurium* were grown as for the *in vitro* secretion assay. Cells were harvested by centrifugation, washed three times in PBS and fixed overnight with 4% formaldehyde in PBS at 4 °C. Cells were again washed three times in PBS, counterstained with 4,6-diamidino-2-phenylindole (Sigma-Aldrich) and 10 mM Nile Red (Sigma-Aldrich), and immobilized on poly-L-lysine (Sigma-Aldrich)-coated coverslips. Covers were mounted in Prolong Diamond (Life Technologies) and sealed with nail polish. Slides were imaged on a DeltaVision Image Restoration Microscope with a × 100 objective (Applied Precision). Images were deconvoluted in Softworx (Applied Precision) and processed identically in ImageJ (NIH) and Photoshop (Adobe).

## Additional information

**Accession codes:** Atomic coordinates have been deposited in the Protein Data Bank under the following deposition codes: 4YX1, SpaO(232–297, SeMet); 4YX5, SpaO(145–213)+SpaO(232–297); 4YX7, SpaO(145–213)+SpaO(232–297)+OrgB(1–30)::T4 lysozyme; 4YXA, SpaO(145–213, SeMet)+SpaO (232–297, SeMet)+OrgB(1–30)::T4 lysozyme (native); 4YXB, FliM(245–334)::FliN(5–137) SeMet; 4YXC, FliM(245–334)::FliN(5–137)+FliH(1–18)::T4 lysozyme. NMR chemical shifts are deposited in the BMRB under ID 26543 (apo-SpaO) and 26546 (APAR-bound SpaO).

**How to cite this article:** Notti, R.Q. *et al*. A common assembly module in injectisome and flagellar type III secretion sorting platforms. *Nat. Commun.* 6:7125 doi: 10.1038/ncomms8125 (2015).

## Supplementary Material

Supplementary Information Supplementary Figures 1-10

## Figures and Tables

**Table 1:** Data collection and refinement statistics for injectisome structures.

	**SpaO(232–297, SeMet)**	**SpaO(145–213, SeMet)+SpaO(232–297, SeMet)**	**SpaO(145–213)+SpaO(232–297)**	**SpaO(145–213)+SpaO(232–297)+OrgB(1–30)::lysozyme**	**SpaO(145–213, SeMet)+SpaO(232–297, SeMet)+OrgB(1–30)::lysozyme**
*Data collection*					
Space group	P 2_1_	P 4_1_ 2_1_ 2	P 4_1_ 2_1_ 2	P 2_1_	P 2_1_
Cell dimensions					
*a*, *b*, *c* (Å)	35, 41.27, 48	66.38, 66.38, 95.21	65.76, 65.76, 95.65	62.092, 89.07, 62.092	62.88, 88.5, 63.32
*α*, *β*, *γ* (°)	90, 103.92, 90	90, 90, 90	90, 90, 90	90, 114.94, 90	90, 116.07, 90
Resolution (Å)	31.26–1.35 (1.37–1.35)	46.94–3.00 (3.18–3.00)	38.68–2.9 (3.08–2.9)	47.59–2.0 (2.05–2.0)	45.8–2.35 (2.43–2.35)
*R*_merge_	0.146 (1.281)	0.221 (1.463)	0.166 (1.447)	0.102 (0.530)	0.088 (0.617)
*I*/σ*I*	8.6 (2.1)	11.8 (3.1)	14.4 (2.7)	10.5 (3.2)	12.8 (2.6)
CC_1/2_	0.994 (0.750)	0.989 (0.839)	0.996 (0.856)	0.994 (0.828)	0.997 (0.816)
Completeness (%)	99.7 (100)	100 (100)	99.3 (99.3)	99.5 (99.8)	99.0 (99.6)
Redundancy	7.0 (7.1)	25.6 (27.2)	24.6 (26.2)	5.1 (5.2)	6.6 (6.6)
					
*Refinement*					
No. of reflections	29,246		4,964	41,183	25,740
*R*_work_/*R*_free_	0.1724/0.2053		0.2085/0.2795	0.1571/0.2096	0.1984/0.2618
No. of atoms	1,286		1,024	5,769	4,940
Protein	1,062		1,023	5,112	4,818
Ligand/ion	2		1	0	0
Water	222		0	657	122
*B* factors					
Protein	14.70		74.20	33.10	46.90
Ligand/ion	14.20		105.00		
Water	32.10			39.60	45.60
r.m.s.d.					
Bond lengths (Å)	0.007		0.010	0.008	0.011
Bond angles (°)	1.09		1.33	1.16	1.46

SeMet, selenomethionine.

**Table 2:** Data collection and refinement statistics for flagellar structures.

	**FliM(245–334)::FliN(5–137), SeMet**	**FliM(245–334)::FliN(5–137)+FliH(1–18)::lysozyme**
*Data collection*		
Space group	P 2_1_ 2_1_ 2_1_	P 2_1_ 2_1_ 2_1_
Cell dimensions		
*a*, *b*, *c* (Å)	75.15, 81.50, 89.96	43.21, 76.37, 119.4
*α*, *β*, *γ* (°)	90, 90, 90	90, 90, 90
Resolution (Å)	57.67–2.56 (2.67–2.56)	64.33–2.30 (2.38–2.30)
*R*_merge_	0.097 (1.215)	0.070 (0.923)
*I*/σ*I*	18.5 (2.7)	20.2 (2.6)
CC_1/2_	0.999 (0.814)	0.999 (0.811)
Completeness (%)	100 (100)	99.8 (99.8)
Redundancy	13.8 (14.3)	12.9 (12.9)
		
*Refinement*		
No. of reflections	18,372	18,174
*R*_work_/*R*_free_	0.2175/0.2593	0.1967/0.2620
No. of atoms	2,633	2,739
Protein	2,605	2,668
Ligand/ion	5	0
Water	23	71
*B* factors		
Protein	68.30	69.70
Ligand/ion	73.40	
Water	64.50	65.00
r.m.s.d.		
Bond lengths (Å)	0.010	0.009
Bond angles (°)	1.31	1.15

SeMet, selenomethionine.

## References

[b1] Galan J. E., Lara-Tejero M., Marlovits T. C. & Wagner S. Bacterial type III secretion systems: specialized nanomachines for protein delivery into target cells. Annu. Rev. Microbiol. 68, 415–438 (2014). 25002086 10.1146/annurev-micro-092412-155725 PMC4388319

[b2] Aldridge P. & Hughes K. T. Regulation of flagellar assembly. Curr. Opin. Microbiol. 5, 160–165 (2002). 11934612 10.1016/s1369-5274(02)00302-8

[b3] Kawamoto A. . Common and distinct structural features of Salmonella injectisome and flagellar basal body. Sci. Rep. 3, 3369 (2013). 24284544 10.1038/srep03369 PMC3842551

[b4] Lara-Tejero M., Kato J., Wagner S., Liu X. & Galan J. E. A sorting platform determines the order of protein secretion in bacterial type III systems. Science 331, 1188–1191 (2011). 21292939 10.1126/science.1201476 PMC3859126

[b5] Bzymek K. P., Hamaoka B. Y. & Ghosh P. Two translation products of Yersinia yscQ assemble to form a complex essential to type III secretion. Biochemistry 51, 1669–1677 (2012). 22320351 10.1021/bi201792p PMC3289748

[b6] Fadouloglou V. E. . Structure of HrcQB-C, a conserved component of the bacterial type III secretion systems. Proc. Natl Acad. Sci. USA 101, 70–75 (2004). 14694203 10.1073/pnas.0304579101 PMC314140

[b7] Brown P. N., Mathews M. A., Joss L. A., Hill C. P. & Blair D. F. Crystal structure of the flagellar rotor protein FliN from Thermotoga maritima. J. Bacteriol. 187, 2890–2902 (2005). 15805535 10.1128/JB.187.8.2890-2902.2005 PMC1070373

[b8] Zhao R., Pathak N., Jaffe H., Reese T. S. & Khan S. FliN is a major structural protein of the C-ring in the Salmonella typhimurium flagellar basal body. J. Mol. Biol. 261, 195–208 (1996). 8757287 10.1006/jmbi.1996.0452

[b9] Morita-Ishihara T. . Shigella Spa33 is an essential C-ring component of type III secretion machinery. J. Biol. Chem. 281, 599–607 (2006). 16246841 10.1074/jbc.M509644200

[b10] Hu B. . Visualization of the type III secretion sorting platform of Shigella flexneri. Proc. Natl Acad. Sci. USA 112, 1047–1052 (2015). 25583506 10.1073/pnas.1411610112 PMC4313800

[b11] Diepold A., Kudryashev M., Delalez N. J., Berry R. M. & Armitage J. P. Composition, formation, and regulation of the cytosolic c-ring, a dynamic component of the type III secretion injectisome. PLoS Biol. 13, e1002039 (2015). 25591178 10.1371/journal.pbio.1002039 PMC4295842

[b12] Bergeron J. R. . The modular structure of the inner-membrane ring component PrgK facilitates assembly of the type III secretion system basal body. Structure 23, 161–172 (2015). 25533490 10.1016/j.str.2014.10.021

[b13] Mizusaki H., Takaya A., Yamamoto T. & Aizawa S. Signal pathway in salt-activated expression of the Salmonella pathogenicity island 1 type III secretion system in Salmonella enterica serovar Typhimurium. J. Bacteriol. 190, 4624–4631 (2008). 18441068 10.1128/JB.01957-07 PMC2446791

[b14] Minamino T. . Roles of the extreme N-terminal region of FliH for efficient localization of the FliH-FliI complex to the bacterial flagellar type III export apparatus. Mol. Microbiol. 74, 1471–1483 (2009). 19889085 10.1111/j.1365-2958.2009.06946.x

[b15] Kihara M., Francis N. R., DeRosier D. J. & Macnab R. M. Analysis of a FliM-FliN flagellar switch fusion mutant of Salmonella typhimurium. J. Bacteriol. 178, 4582–4589 (1996). 8755888 10.1128/jb.178.15.4582-4589.1996 PMC178227

[b16] Bai F. . Assembly dynamics and the roles of FliI ATPase of the bacterial flagellar export apparatus. Sci. Rep. 4, 6528 (2014). 25284201 10.1038/srep06528 PMC4185386

[b17] Hara N., Morimoto Y. V., Kawamoto A., Namba K. & Minamino T. Interaction of the extreme N-terminal region of FliH with FlhA is required for efficient bacterial flagellar protein export. J. Bacteriol. 194, 5353–5360 (2012). 22843851 10.1128/JB.01028-12 PMC3457192

[b18] Sievers F. . Fast, scalable generation of high-quality protein multiple sequence alignments using Clustal Omega. Mol. Syst. Biol. 7, 539 (2011). 21988835 10.1038/msb.2011.75 PMC3261699

[b19] Wallace I. M., O'Sullivan O., Higgins D. G. & Notredame C. M-Coffee: combining multiple sequence alignment methods with T-Coffee. Nucleic Acids Res. 34, 1692–1699 (2006). 16556910 10.1093/nar/gkl091 PMC1410914

[b20] Buchan D. W., Minneci F., Nugent T. C., Bryson K. & Jones D. T. Scalable web services for the PSIPRED Protein Analysis Workbench. Nucleic Acids Res. 41, W349–357 (2013). 23748958 10.1093/nar/gkt381 PMC3692098

[b21] Shoichet B. K., Baase W. A., Kuroki R. & Matthews B. W. A relationship between protein stability and protein function. Proc. Natl Acad. Sci. USA 92, 452–456 (1995). 7831309 10.1073/pnas.92.2.452 PMC42758

[b22] Kaniga K., Bossio J. C. & Galan J. E. The Salmonella typhimurium invasion genes invF and invG encode homologues of the AraC and PulD family of proteins. Mol. Microbiol. 13, 555–568 (1994). 7997169 10.1111/j.1365-2958.1994.tb00450.x

[b23] Bhaskaran S. S. & Stebbins C. E. Structure of the catalytic domain of the Salmonella virulence factor SseI. Acta Crystallogr. D. Biol. Crystallogr. 68, 1613–1621 (2012). 23151626 10.1107/S0907444912039042 PMC3498931

[b24] Battye T. G., Kontogiannis L., Johnson O., Powell H. R. & Leslie A. G. iMOSFLM: a new graphical interface for diffraction-image processing with MOSFLM. Acta Crystallogr. D. Biol. Crystallogr. 67, 271–281 (2011). 21460445 10.1107/S0907444910048675 PMC3069742

[b25] Evans P. R. & Murshudov G. N. How good are my data and what is the resolution? Acta Crystallogr. D. Biol. Crystallogr. 69, 1204–1214 (2013). 23793146 10.1107/S0907444913000061 PMC3689523

[b26] Karplus P. A. & Diederichs K. Linking crystallographic model and data quality. Science 336, 1030–1033 (2012). 22628654 10.1126/science.1218231 PMC3457925

[b27] Adams P. D. . The Phenix software for automated determination of macromolecular structures. Methods 55, 94–106 (2011). 21821126 10.1016/j.ymeth.2011.07.005 PMC3193589

[b28] Emsley P., Lohkamp B., Scott W. G. & Cowtan K. Features and development of Coot. Acta Crystallogr. D. Biol. Crystallogr. 66, 486–501 (2010). 20383002 10.1107/S0907444910007493 PMC2852313

[b29] Thorn A. & Sheldrick G. M. ANODE: anomalous and heavy-atom density calculation. J. Appl. Crystallogr. 44, 1285–1287 (2011). 22477786 10.1107/S0021889811041768 PMC3246834

[b30] McNicholas S., Potterton E., Wilson K. S. & Noble M. E. Presenting your structures: the CCP4mg molecular-graphics software. Acta Crystallogr. D. Biol. Crystallogr. 67, 386–394 (2011). 21460457 10.1107/S0907444911007281 PMC3069754

[b31] Salzmann M., Pervushin K., Wider G., Senn H. & Wuthrich K. TROSY in triple-resonance experiments: new perspectives for sequential NMR assignment of large proteins. Proc. Natl Acad. Sci. USA 95, 13585–13590 (1998). 9811843 10.1073/pnas.95.23.13585 PMC24862

[b32] Sattler M., Schleucher J. & Griesinger C. Heteronuclear multidimensional NMR experiments for the structure determination of proteins in solution employing pulsed field gradients. Prog. Nucl. Magn. Reson. Spectrosc. 34, 93–158 (1999).

[b33] Keller R. L. J. The Computer Aided Resonance Assignment Tutorial CANTINA (2004).

[b34] Shen Y., Delaglio F., Cornilescu G. & Bax A. TALOS+: a hybrid method for predicting protein backbone torsion angles from NMR chemical shifts. J. Biomol. NMR 44, 213–223 (2009). 19548092 10.1007/s10858-009-9333-z PMC2726990

