# Supplementary material for: A common assembly module in injectisome and flagellar type III secretion sorting platforms
Source: Nat Commun. 2015 May 21;6:7125. doi: 10.1038/ncomms8125 (PMC4443714; doi:10.1038/ncomms8125)
Supplement: Supplementary Information — Supplementary Figures 1-10 [file ncomms8125-s1.pdf]

Supplementary Figure 1

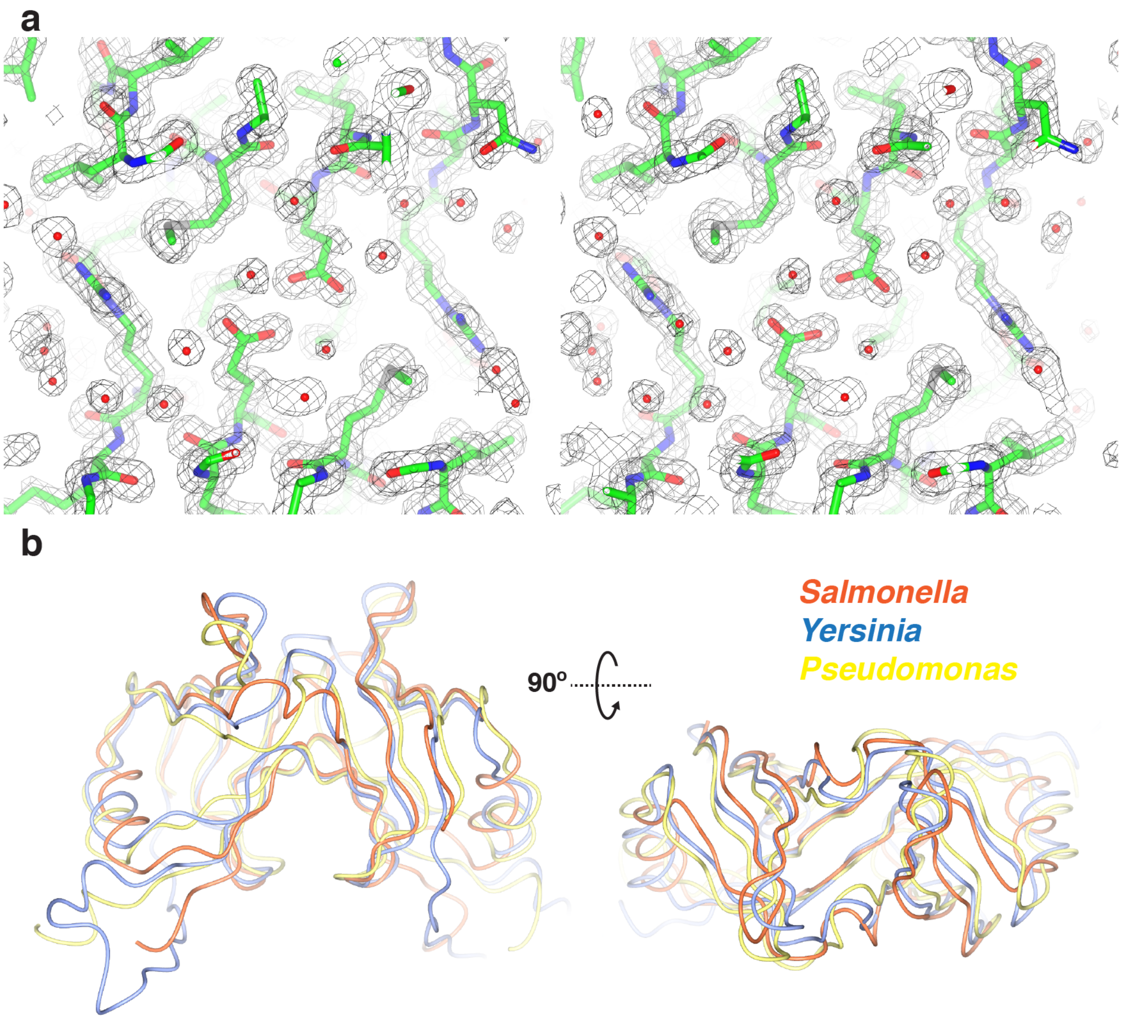

Analysis of the SpaO SPOA2,2 structure. (a) Stereoimage of a selected region of the 2Fo-Fc electron density map for the 1.35Å resolution SpaO(232-297) structure. The map is contoured to 1σ. (b) Comparison of the SpaO(232-297) homodimer structure (orange) with its homologues in the PDB (*Yersinia*, 3UEP, purple, 2.24Å RMSD; *Pseudomonas*, 1O9Y, yellow, 3.05Å RMSD).

Supplementary Figure 2

a

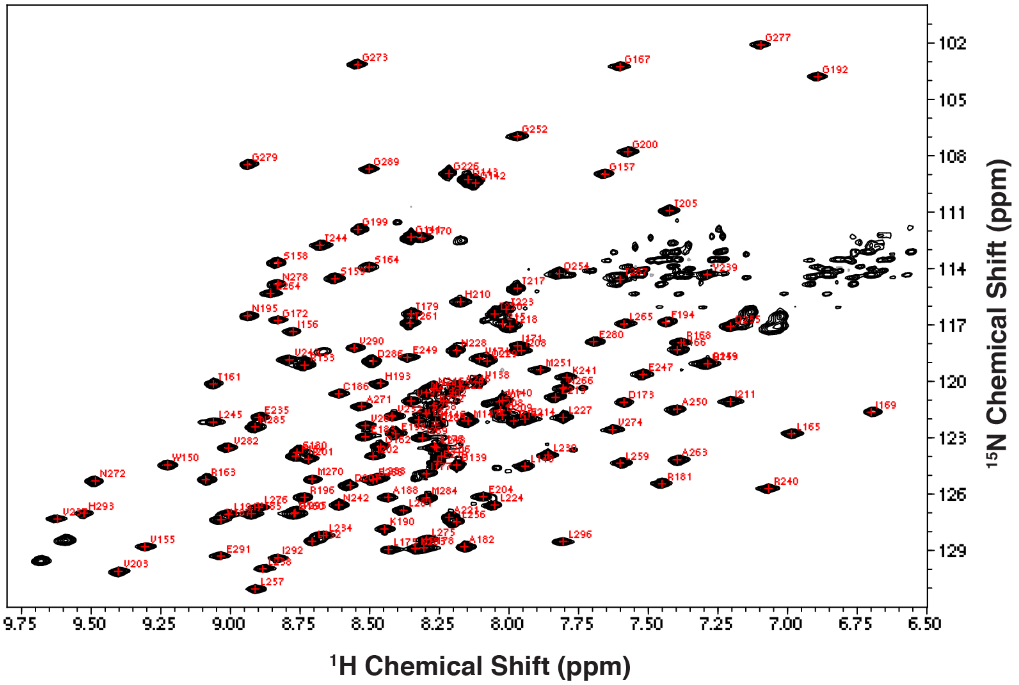

b

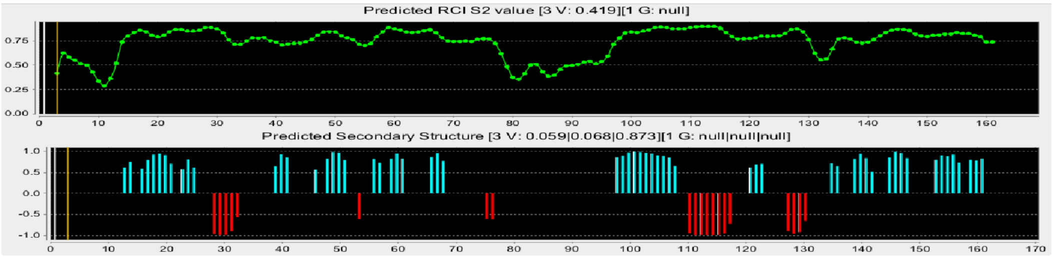

NMR analysis of SpaO SPOA1,2. (a) ^{15}N-TROSY for SpaO(140-297) annotated with backbone amide resonance assignments in red. (b) TALOS+ prediction of SpaO(140-297) order (green) and secondary structure (blue, strand; red, helix). The overall impression is one of two domains connected by a flexible linker.

Supplementary Figure 3

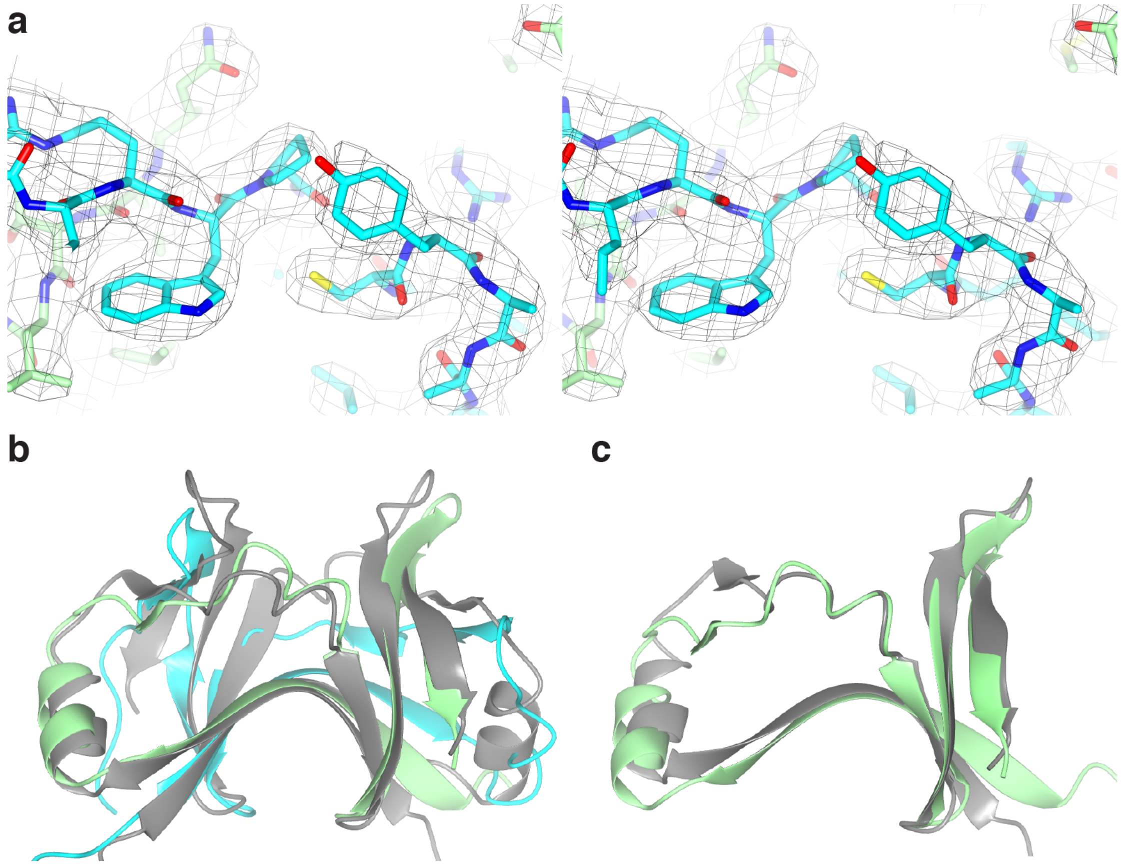

Analysis of the SpaO SPOA1,2 structure. (a) Stereoimage of a selected region of the 2Fo-Fc electron density map for the 2.9Å SpaO(145-213)+SpaO(232-297) structure. The map is contoured to 1σ and clipped to within 2Å of the peptide for clarity. (b) Superposition of SPOA1,2 (cyan and green, respectively) on the SPOA2 homodimer (grey) structure (2.47Å RMSD). (c) Superposition of the SpaO SPOA2 from the homodimer (grey) and SPOA1,2 (green) structures (1.67Å RMSD).

Supplementary Figure 4

a

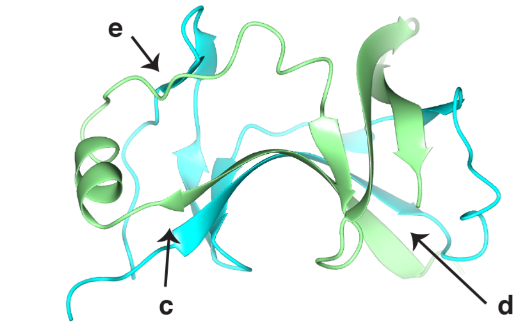

b

SPOA1	SPOA2	Dist.(Å)
W150	V243	2.7
L152	K241	3.1
T161	V232	3.5
I201	L257	2.8
I201	L259	3.9

c d e

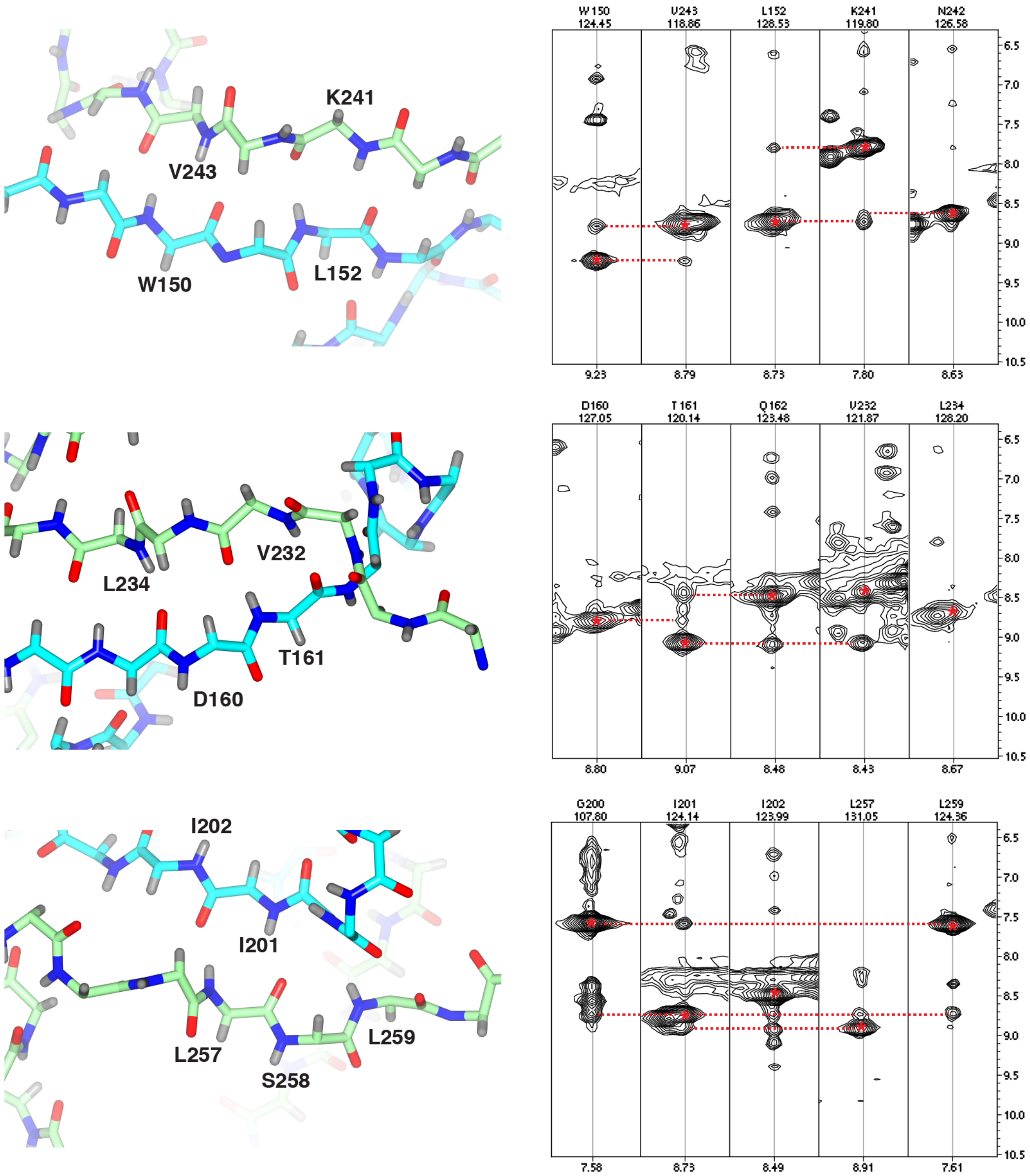

SpaO SPOA1 and SPOA2 interact in solution. (a) Schematic illustration of the SPOA1,2 crystal structure with regions for further analysis indicated. (b) Protons were modeled on the SPOA1,2 structure using ReadySet in Phenix. Inter-proton distances are specified for selected inter-domain long-range amide proton correlations observed in the ^{15}N-NOESY-HSQC spectrum. (c-e) Protonated backbone models for the regions specified in (a) are shown at left with residues of interest noted. In the ^{15}N-NOESY-HSQC spectra at right, the diagonal-peaks are indicated by red asterisks and the cross-peaks by red dotted lines.

Supplementary Figure 5

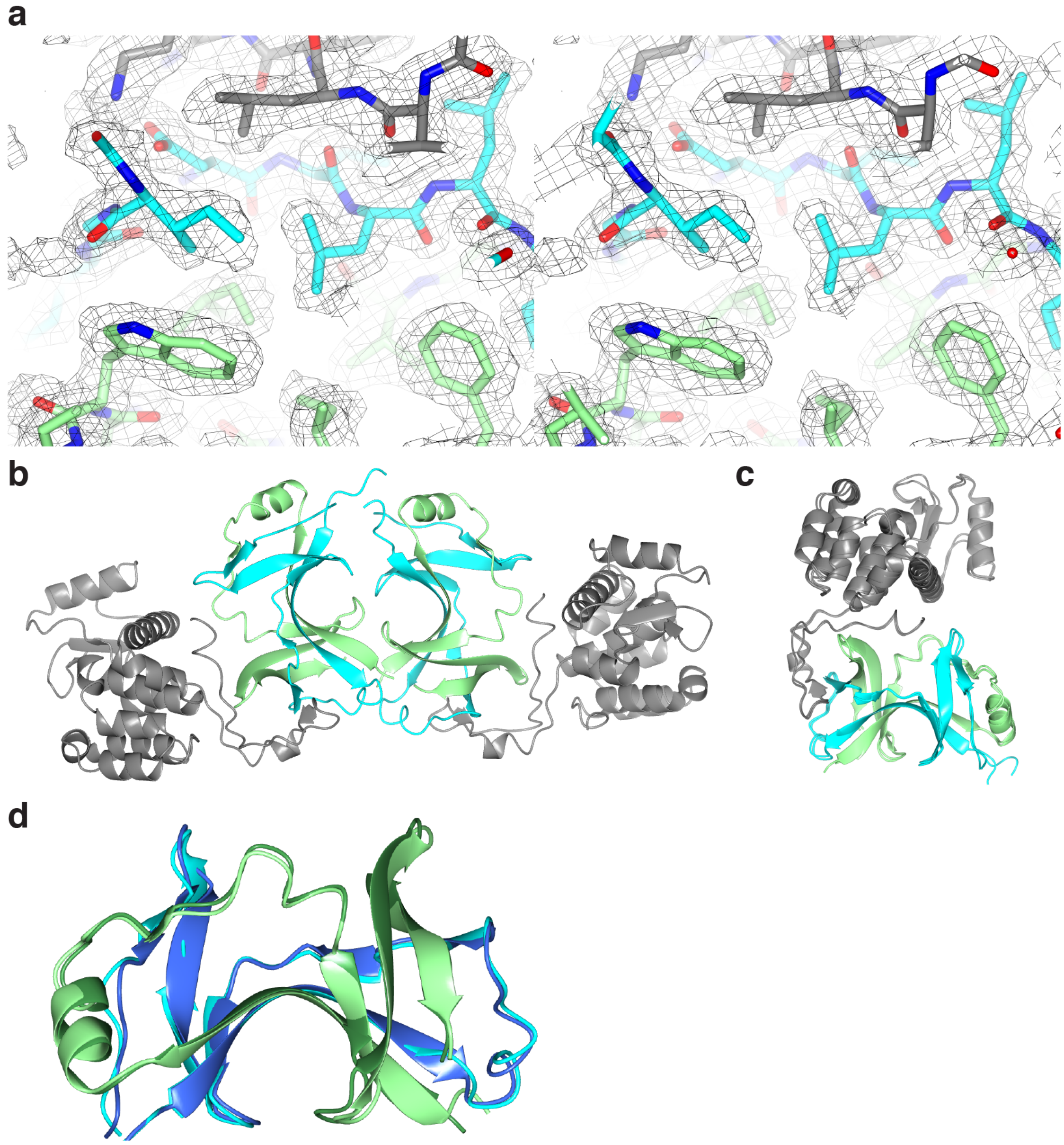

Analysis of the SpaO-OrgB SPOA1,2-APAR structure. (a) Stereoimage of a selected region of the 2Fo-Fc electron density map for the 2.0Å SpaO(145-213, cyan)+SpaO(232-297, green)+OrgB(1-30)::T4 lysozyme (grey) structure. The map is contoured to 1σ. (b) The asymmetric unit of the SpaO(145-213, cyan)+SpaO(232-297, green)+OrgB(1-30)::T4 lysozyme (grey) crystal contains two copies of the SpaO-OrgB complex. (c) Superposition of the two constituents in the asymmetric unit in the SpaO(145-213,

cyan)+SpaO(232-297, green)+OrgB(1-30)::T4 lysozyme crystal showing their architectural similarity (0.81Å RMSD). (d) Superposition of the apo- (cyan/light green) and APAR-bound (blue/dark green) forms of SpaO (1.01Å RMSD).

Supplementary Figure 6

a

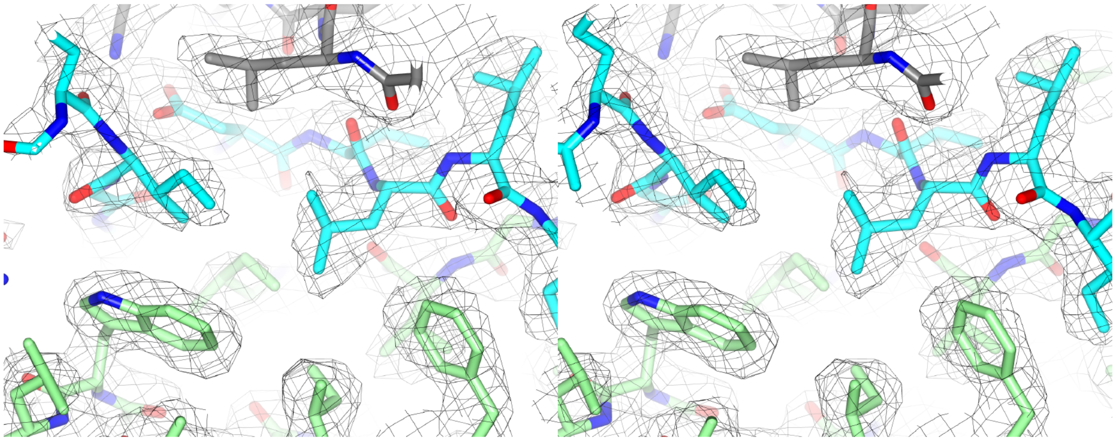

b

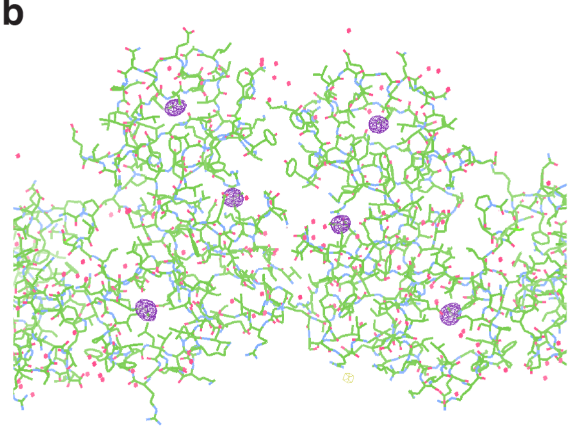

Empirical confirmation of the SpaO-OrgB SPOA1,2-APAR structure. (a) Stereoimage of a selected region of the 2Fo-Fc electron density map for the 2.35Å SpaO(145-213, SeMet, cyan)+SpaO(232-297, SeMet, green)+OrgB(1-30)::T4 lysozyme (native, grey) structure. The map is contoured to 1σ. This structure was solved by molecular replacement using the native structure, and the anomalous signal from SpaO-incorporated SeMet was analyzed in ANODE. (b) ANODE analysis of the anomalous signal in SpaO-OrgB::Lysozyme complex generated by SeMet substitution in SpaO (but not OrgB::T4 lysozyme). The anomalous signal (violet, contoured at 8e/Å^3) localizes to the three structured SeMet in each copy of SpaO(232-297), providing empirical

confirmation of the SpaO-OrgB model coordinates and stoichiometry. The image was created in COOT.

Supplementary Figure 7

```
SpaO    M---SLRVR---------QIDRREWLLAQTATECQRHGREA--TL-------EYPTRQGMWVRLSDAEKRWSAWI
Spa33   ----MLRIKHFDANEKLQILYAKQLCERFSIQTFKNKFTGSESLVTLTSVCGDWVIRIDTLSFLKKKYEVFSGFS
YscQ    M--SLLTLP---------QAKLSELSLRQRLSHYRQNYLWEEGKLELT--VSEPPSSLNCILQLQWKGTHFTLYC
PscQ    MNGADLDLP---------LASRAELDLQRRLARCRRHYVGNALQARLD--IAQAAPDVDLELSLAWDGLPLRFLC

cons         * :               :          :.:               :     .    *


SpaO    KPGDWLEHVSPALAGAAVSAGAEHLVVPWLAATERPFELPVPHLSCRRLCVENPVPGSALP--EGKLLHIMSDRG
Spa33   TQESLLH-----LSKCVFIESSSVFSIPELSDKITFR-------ITNEIQYATTG--SHLCCFS-------SSLG
YscQ    FGDDLANWLTPDLLGAPFSTLPKELQLALLERQTVFL----PKLVCNDIATASLS--VTQPLLSLR-LSRDNAHI
PscQ    QAPALARWLAPNLQEAAFASLPAALQLALLEREGNVF----PGLVWYGLSPAQPR--AAMGL-RLS-LERDDQRL

cons          .     *  . .   .  : :. *                  :                      .


SpaO    GLWFEHLPE-LPAVGGGRPKMLRWPLRFVIG----SSDTQRSLLGRIGIGDVLLIRTSRA-----EVYCYAKKLG
Spa33   IIYFDKMPV---LRNQVSLDLLHHLLEFCLG----SSNVRLATLKRIRTGDIIIVQKLYNL-----LLCNQVIIG
YscQ    SFWLTSAEA-LFALLPARPNSERIPLPILLSLRWHKVYLTLDEVDSLRLGDVLLAPEGSGPNSPVLAYVGENPWG
PscQ    ALWLDGDPATLLARLPPRPSAQRLAIPLRLSLQWPGLPLEAGELRTLEPGDLLLLPAGHRPDAALLGVLEGRPWA

cons     :::               .  :  : :.            :  :  **:::                    .


SpaO    HFNRVEGGIIVETLDIQHIEEENNTTETAETLPGLNQLPVKLEFVLYRKNVTLAELEAMGQQQLLSLPTNAELNV
Spa33   DYIVNDNN--EAKINLSESNGESDHTEVSLALFNYDDINVKVDFILLEKKMTINELKMYVENELFKFPDDIVKHV
YscQ    YFQLQSNK--LEFIGMSHESDELN----PKPLTDLNQLPVQVSFEVGRQILDWHTLTSLEPGSLIDLTTPVDGEV
PscQ    RCQLHSTQ--LELLDMHDTPSLAD----SEDLHELDQLPIPVSFPVGRRTLDLHTLSTLQPGSLLDLDCALDGEV

cons         .       :.: .     :    .  *   ::: : :.* : .: :    *      .*:.:      .*


SpaO    EIMANGVLLGNGELVQMNDTLGVEIHEWLSESGNGE
Spa33   NIKVNGSLVGHGELVSIEDGYGIEISSWMVK----E
YscQ    RLLANGRLLGHGRLVEIQGRLGVRIERLTEVTI--S
PscQ    RILANQRCLGIGELVRLQDRLGVRVTRLFGHDE--A

cons    .: .*   :* * ** ::.  *:.:
```

Full M-COFFEE alignment of SpaO and its homologues. Excerpted regions shown in Fig. 4a are highlighted by dashed boxes.

Supplementary Figure 8

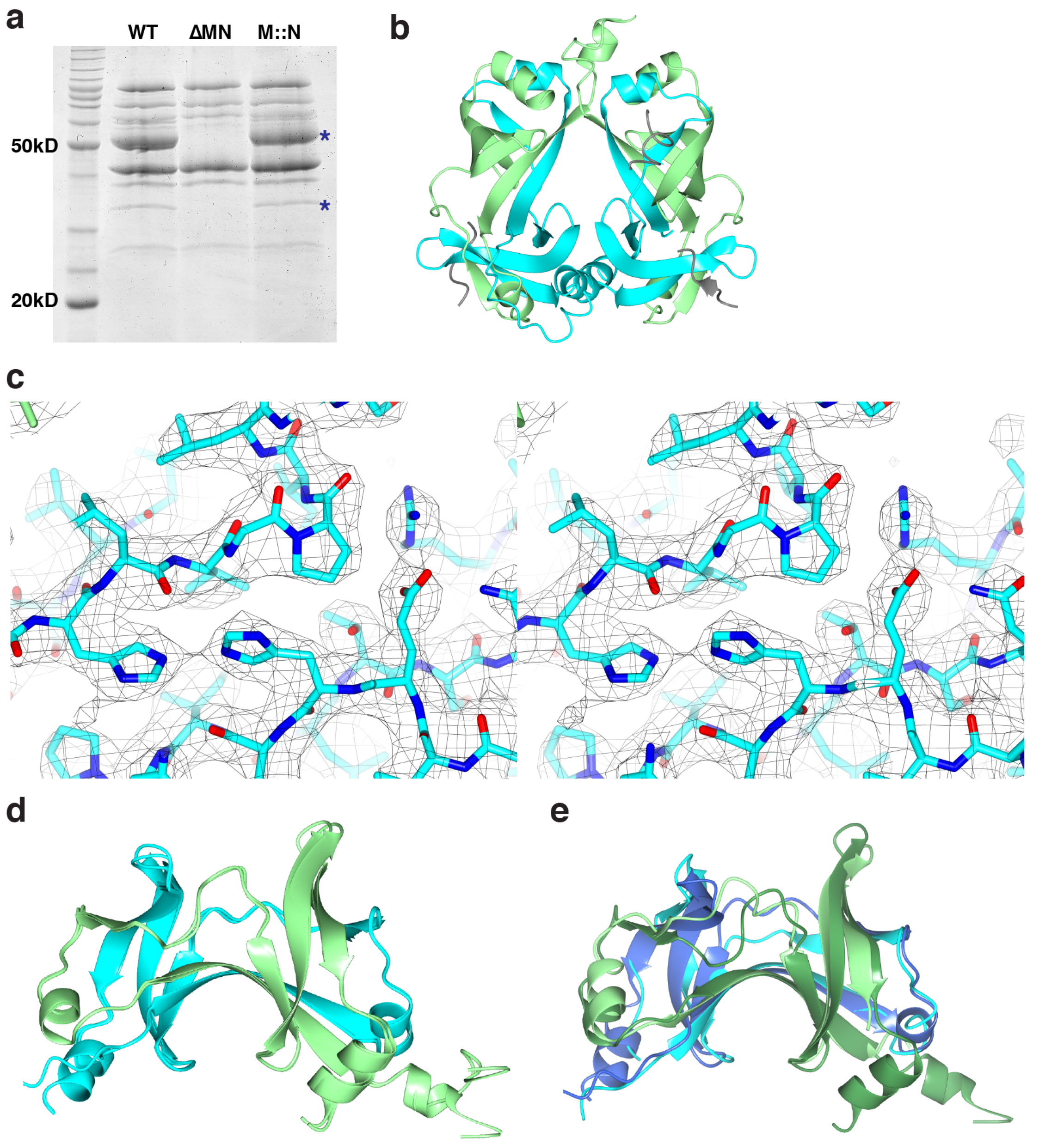

Analysis of the FliM-FliN fusion structure. (a) FliM-FliN fusion is compatible with flagellar secretory function. Coomassie stained PAGE of culture supernatants from *S. typhimurium* with the indicated genotype (WT, wild-type; ΔMN, deletion of *fliM* and *fliN*; M::N, FliM-FliN(5-137) fusion). Flagellar specific secretory products (lost in the *fliM/fliN* deletion background) are present in the FliM-FliN fusion strain (blue asterisks highlight

FliC and FlgL, data shown are representative of three experiments). The FliM::FliN fusion brings FliN residue 5 in frame with full-length FliM; both Met1 and Met4 of FliN are deleted to prevent spurious translation of free FliN. (b-e) Analysis of the FliM::FliN fusion crystal structure. (b) Asymmetric unit of the FliM(245-334, cyan)::FliN(5-137, green) crystal. Noncontiguous modeled peptide fragments are shown in grey. (c) Stereoimage of a selected region of the 2Fo-Fc electron density map for the 2.56Å FliM(245-334, cyan)::FliN(5-137, green) structure. The map is contoured to 1σ. (d) Superposition of the two constituents of the asymmetric unit (0.56Å RMSD). (e) Superposition of apo-SpaO SPOA1,2 (light blue and green, respectively) with apo-FliM::FliN (dark blue and green, respectively; 2.28Å RMSD).

Supplementary Figure 9

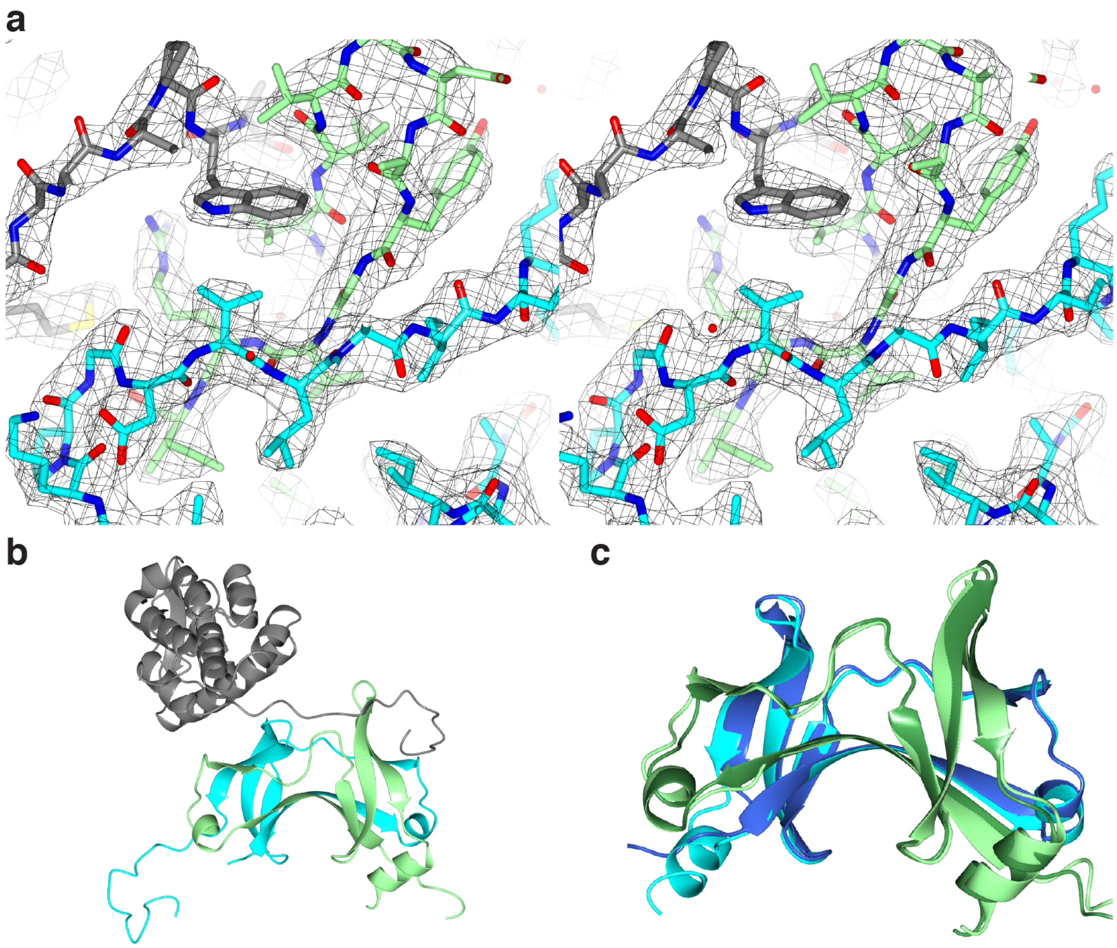

Analysis of the FliM-FliN-FliH crystal structure. (a) Stereoimage of the 2Fo-Fc electron density map for the 2.30Å FliM(245-334, cyan)::FliN(5-137, green) + FliH(1-18)::T4 lysozyme (grey) structure. The map is contoured to 1σ and highlights the FliH W7 binding region. (b) The crystallographic asymmetric unit, colored as in (a). (c) Superposition of apo- (cyan/green) and APAR-bound (blue/dark green) FliM::FliN (1.11Å RMSD).

Supplementary Figure 10

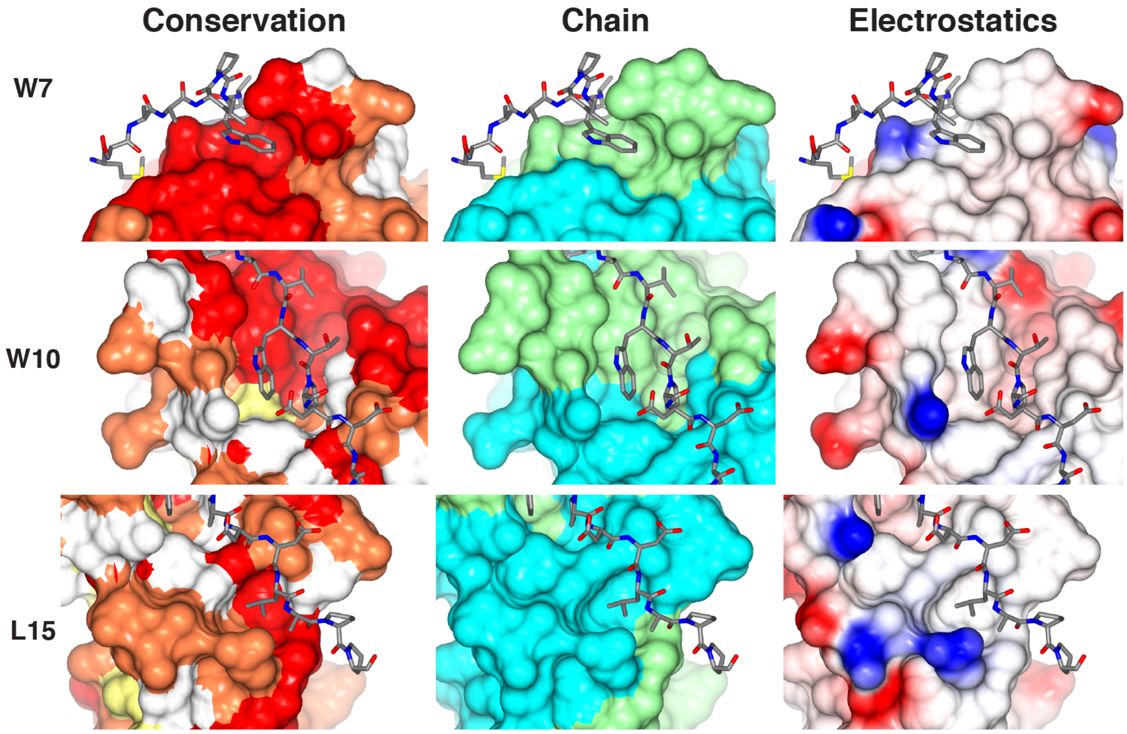

APAR-binding regions on FliM-FliN are well conserved. The binding pockets for FliH W7, W10, and L15 on FliM-FliN are shown as surface renderings. On the left, the surface is color coded by residue conservation across *S. typhimurium, S. flexneri, Y. enterocolica,* and *P. aeruginosa*. Red indicates 100% identity. Orange, yellow, and white indicate high, medium, and low conservation by M-COFFEE multiple sequence alignment. In the middle, the same surface is shown but color-coded by chain: FliM is cyan, FliN is green. On the right, the surface is color coded by electrostatic potential (red, negative; blue, positive).
